# *figsimR*: An R Package for Simulating Fig–Wasp Community Dynamics

**DOI:** 10.1002/ece3.74018

**Published:** 2026-07-20

**Authors:** Yiyi Dong, Simon T. Segar, Qingbei Weng

**Affiliations:** ^1^ Qiannan Normal University for Nationalities Duyun China; ^2^ School of Forest, Fisheries, & Geomatics Sciences University of Florida Gainesville Florida USA; ^3^ Agriculture and Environment Department Harper Adams University Newport UK; ^4^ School of Life Sciences Guizhou Normal University Guiyang China

**Keywords:** community assembly, fig wasps, mechanistic model, simulation, species interaction

## Abstract

Fig–fig wasp systems represent a widely applicable but underutilized framework for community assembly research, yet their multi‐trophic structure and species interactions substantially increase the complexity of community simulation. We present figsimR, an open‐source, modular R package that implements fig wasp community simulation via a single high‐level function, simulate_figwasp_community(), with controllable mechanisms and reproducible workflows. The simulation runs at the individual‐fig level and records species‐specific wasp activity and outcomes, including but not limited to egg production, host‐specific parasitism, final fig wasp emergence, seed production, flower availability, and ovule‐resource use within each fig. The package also provides functions for calculating commonly used diversity and community‐summary metrics, allowing fig‐level community outputs to support downstream comparison and model diagnostics. We demonstrate a typical workflow—parameter setup, simulation, and metric calculation—on fig wasp communities associated with *Ficus racemosa* as a worked example using an agent‐based model that integrates documented biological traits and available data for this system. We also show how the package can be used to assess a baseline model's goodness‐of‐fit and to dissect the asymmetric contributions of individual processes (e.g., ovule‐layer stratification and alternative fig‐retention rules, including host‐sanction and sink strength) using the built‐in “mechanism knockout” framework (allowing to quantify the contribution for each mechanism in community assembly). Including both mean fit and variance coverage as joint diagnostics, reveals mean–variance trade‐offs across mechanisms. Although the worked example is parameterized for *F. racemosa* in this study, figsimR is not restricted to a single *Ficus* species. Users can adapt the framework to other *Ficus*–fig wasp systems by supplying necessary biological parameters. figsimR, therefore, provides a reproducible and extensible tool for evaluating how far known biological mechanisms can explain observed community properties.

## 1 Introduction

Fig–fig wasp mutualisms, distributed across tropical and sub‐tropical habitats, represent highly diverse species interactions across multiple trophic levels and offer a compact, bounded and well replicated system for studying community dynamics (Weiblen 2002; Cook and Rasplus 2003). However, within a given fig‐fig wasp system, species interactions are typically complex. Interspecific differences in oviposition timing and spatial niches, flower competition, and host‐dependent parasitism make simulator development challenging (Weiblen 2002; Kjellberg et al. 2005; Zhang et al. 2006; Herre et al. 2008; Wang et al. 2008; Cook and Segar 2010; Jandér and Herre 2010), because these key parameters are often hard to quantify. At present, therefore, no ready‐to‐use model has been developed to simulate complicated fig wasp communities. Such a model is useful because it provides a mechanism‐explicit baseline for generating null expectations and for comparing observed communities against simulated predictions, in terms of both mean fit and variance coverage. Given that fig wasp communities are increasingly being used as models for studying community assembly and climate change (Aung et al. 2022), it is a priority to effectively test predictions in silico.

To address this gap, some researchers have used “null” models or contrasted communities via resampling‐based null expectations or process‐based models, including lottery and population‐dynamical frameworks (Duthie et al. 2014; Wang et al. 2015; Wang, Yang, et al. 2020; Souza et al. 2025). These studies have successfully identified nonrandom patterns in fig wasp community assembly; however, they often provide limited insight into two fundamental questions: (i) what biological mechanisms generate these patterns, and (ii) to what extent are they shared across fig wasp communities associated with different fig species? For instance, a consumer‐resource model, based on population dynamics of each fig wasp species, was developed to simulate community structure and examine community stability under parameter perturbations (Wang, Yang, et al. 2020). The lottery‐storage model addresses a related question: given competitive exclusion, how can nonpollinating fig wasps with similar niches co‐exist in the same fig (Duthie et al. 2014)? Again, in many of these models, the parameter sets and mechanisms are fixed, thereby limiting users' ability to adjust parameters and assumptions, for example, by switching off processes or incorporating additional processes (e.g., spatially structured oviposition or host sanctions/fruit abortion) to test their effects on community structure, stability, and assembly (Dunn et al. 2008; Jandér and Herre 2010; Cruaud, Jabbour‐Zahab, Genson, Couloux, et al. 2011).

In addition, many models primarily aim to predict conditions for coexistence rather than to support diagnostic comparisons with field observations. Other attempts to compare observed data with null models typically resample observational data (e.g., bootstrap) (Wang et al. 2015; Souza et al. 2025). However, resampling from observed data represents a data‐derived null model that does not explicitly encode biological mechanisms. Mechanism‐explicit tools such as agent‐based models can account for heterogeneity in the temporal and spatial distribution of individuals (Coakley et al. 2006; Zhang and DeAngelis 2020), thus providing a practical solution for simulating fig–wasp communities and computing standard community metrics with minimal setup.

Rather than building a universal fig wasp community model, it is more tractable to simulate the fig wasp community associated with a single fig species. In particular, biological information for each fig wasp species associated with a focal fig species—such as parasitoid relationships, interactions among fig wasps, oviposition behaviors, and oviposition timing windows—is occasionally well documented in the literature for a limited number of intensively studied systems. For instance, *Ficus rubiginosa* Desf. ex Vent., *Ficus hispida* L.f., and *Ficus racemosa* L. are well‐studied fig species for which community composition has been recorded across different habitats, seasons, niches, and regions (Peng et al. 2005; Wang, Yang, Zhao, and Yamg 2005; Wang, Yang, and Yang 2005; Ghara and Borges 2010; Segar et al. 2014; Yadav and Borges 2018; Aung et al. 2022; Jauharlina et al. 2025).

We aim to effectively model community assembly in a well‐studied species of *Ficus*, using figsimR, a modular, agent‐based R package for simulating fig–wasp communities associated with *F. racemosa* . Although we use *F. racemosa* as the worked example here, the framework is general and can be adapted and applied to other fig species when species roles and interaction information are available. The simulation of fig wasp communities at the individual‐fig level is achieved by running a single function, simulate_figwasp_community(), under explicit mechanisms (e.g., arrival stochasticity of fig wasps, spatial partitioning among fig wasps, and host sanctions). The effect of each biological parameter on the community can be assessed by switching modules on or off. Further, the simulated output allows users to compute α‐diversity—richness and Pielou's evenness (Pielou 1966), β‐diversity—Bray‐Curtis and multivariate dispersion (Bray and Curtis 1957; Anderson 2006), and network metrics—nestedness, connectance, and modularity (Dunne et al. 2002; Newman 2006; Almeida‐Neto et al. 2008). The output dataset at fig‐level with a clean format can be used for downstream visualization and comparison.

This application paper outlines figsimR's design conception, including modularity, controllability, and reproducibility. It introduces a minimal workflow. Users set parameters, run simulations, and calculate community metrics on a dataset. We also summarize validation tests, potential applications, and key biological constraints of the *figsimR* package. Here, we conclude with the availability of *figsimR* and directions for extending the package to other fig‐fig wasp systems or similar plant‐insect mutualisms.

## 2 Materials and Methods

### 2.1 *figsimR* Package Design Goals

**FIGURE 1:**
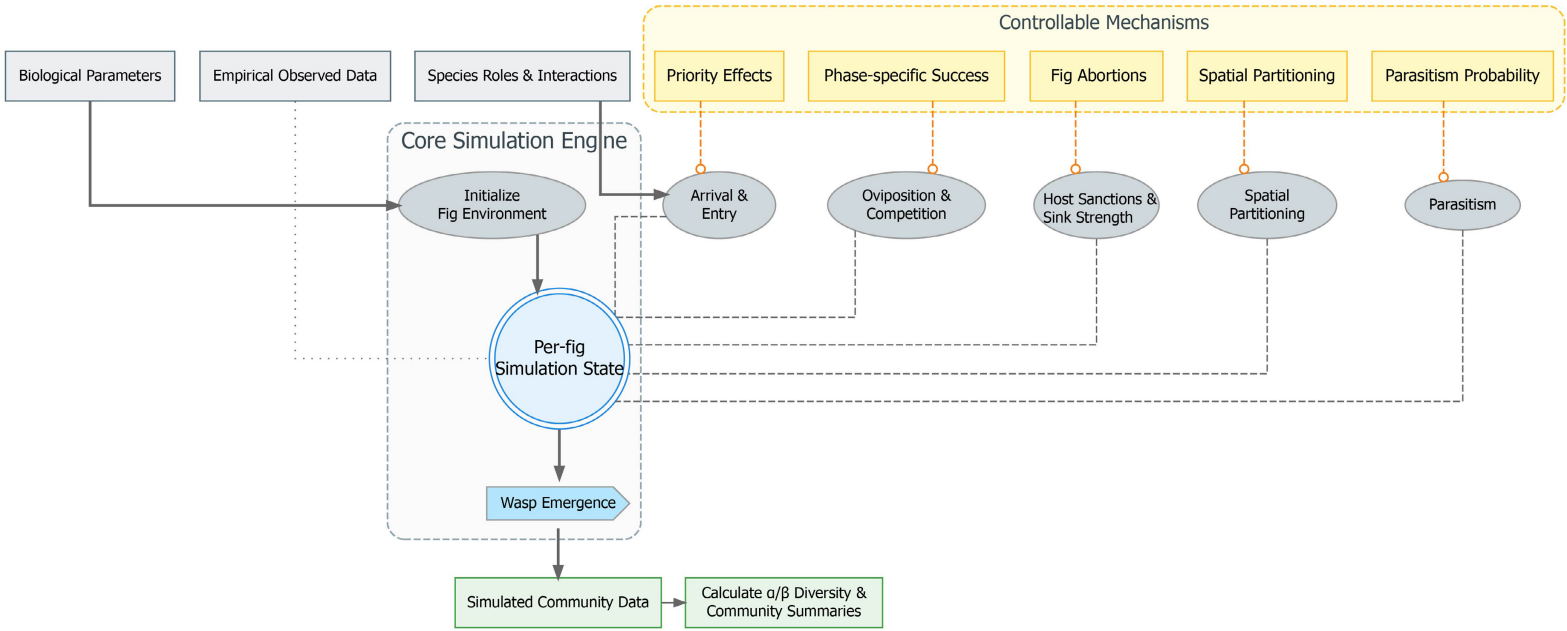
Package workflow illustrating the key steps in fig‐wasp community simulation. Inputs include the records (e.g., average diameter of figs, species list, and their trophic levels) from various documented materials. The function, simulate_figwasp_community(), performs the community composition simulation, integrating available mechanisms, and outputs the tidy dataset at the individual fig level. The simulated data is further used to calculate diversity and community summaries.

*figsimR* is a modular, agent‐based R package for simulating fig‐wasp communities with controllable mechanisms and reproducible outputs (Figure 1). Users can enable or disable specific ecological processes—such as flower resource budgets (Wang et al. 2008), host‐dependent parasitism (Cook and Segar 2010), different fruit abortion theories (Jandér and Herre 2010; Segar et al. 2025), and spatial layering (Ganeshaiah et al. 1995; Kjellberg et al. 2001), without editing core code. All stochastic draws are seed‐controlled, so outputs are tidy and stable, allowing users to identify the biological mechanism resulting in the pattern in observed data by enabling or disabling corresponding modules.

figsimR is not a general‐purpose agent‐based modeling engine, a GUI, or an automatic parameter‐fitting framework. Because empirical datasets at fig‐level remain limited for most fig–fig wasp systems, the current release is dedicated to the simulation of fig–wasp communities associated with *F. racemosa* as a default worked example. However, the simulation framework in the packages is not restricted to *F. racemosa*. Users can customize parameters, such as fig wasp species, functional guilds, and their host‐parasitoid relationships, to adapt the package to other *Ficus*–fig wasp systems when system‐specific data are available.

### 2.2 Fig Wasp Communities Associated With *Ficus racemosa*

Comprehensive details, including community composition records, and species interactions among fig wasp species, and biological traits of figs, have been documented in *F. racemosa* –fig wasp system. While there is genetic variation among populations of *F. racemosa* and some turnover of associated wasp species (Bain et al. 2016; Yu et al. 2019), the trophic structure, and core guilds appear broadly conserved across the studied regions. Fig wasps in this system exhibit clear trophic roles, an obligate pollinator (*Ceratosolen* sp.), two nonpollinating gallers (*Sycophaga mayri, S. testacea*), and three parasitoids (*Apocrypta* sp., *A. westwoodi* , *Sycophaga agraensis*). Among these species temporal entry windows along fig development are also well‐documented (Aung et al. 2022). This background allows us to encode processes transparently and to compare the goodness‐of‐fit of simulated data with observed communities (Weiblen 2002; Cook and Rasplus 2003).

### 2.3 Overview of Model Structure

The model simulated a community of six species across three functional guilds (one pollinator, two gallers, three parasitoids), and their oviposition windows during fig development (syconium development) were integrated in the simulation (Aung et al. 2020, 2022), resulting in temporally and trophically constrained interactions that shape community assembly within each fig. The trophic links and temporal interaction structure among fig wasps were integrated in the model (Figure S1).

The model proceeds in discrete steps corresponding to developmental phases of the fig, with individual wasps stochastically assigned to enter, oviposit, and interact based on species‐specific behavioral traits and ecological rules. The parameters include (but are not limited to) the implementation of fig initialization, wasp entry, egg‐laying behavior, host‐parasitoid interactions, resource constraints, and fig abortion logic (Table 1 and Table S1). In the simulation, “entry” denotes the scheduling of oviposition opportunities (species arrival or oviposition‐attempt events) rather than the physical penetration of wasps into the fig.

**TABLE 1:** Description of key ecological parameters used in figsimR package. The table outlines the parameter's role and how it is implemented in the model. In this framework, an “entrant” (or “entry”) is defined as a reproductive female's arrival at a fig or oviposition‐attempt event.

Parameter group & name format	Description
*Fig & flower resource*	
num_figs	Number of fig simulations to run per replicate (i.e., number of independent communities, default = 1000).
fig_diameter_mean	Mean fig diameter (default = 2.5) used to determine ovule number, in cm. Affects resource availability.
fig_diameter_sd	Standard deviation of fig diameter in cm (assumes normal distribution across figs, default = 1.2).
fig_diameter_min	Minimum allowable fig diameter (default = 1.3). Used to truncate the lower tail of diameter distribution.
fig_diameter_max	Maximum allowable fig diameter (default = 4). Used to truncate the upper tail of diameter distribution.
k	Scaling constant for estimating flower number from fig (default = 300). Controls ovule‐based competition.
alpha	Exponent in the diameter‐flower power‐law used to estimate flower number from fig diameter (default = 1.3).
host_sanction	Threshold parameter (default = 0.8) used in the optional host‐sanction/fig‐abortion module (i.e., host sanction strength). Ranges from 0 (never drop) to 1 (always drop).
use_sink_strength	Logical. If TRUE, compute sink‐strength metrics but do NOT alter legacy drop decision.
sink_w_gall	Numeric [0,1]. Per‐ovule sink weight for galled ovules (default 1.0).
sink_w_seed	Numeric [0,1]. Per‐ovule sink weight for seeds (default 1.5).
p_pollination_per_ovule	Per‐unoccupied‐ovule probability of seed production in the current model implementation (default = 0.98). It represents the efficiency of pollen transfer and directly influences the trade‐off between seed production and wasp reproduction for the finite pool of ovules.
*Wasp arrival & egg deposition dynamics*	
entry_mu	Mean number of each species arrival or oviposition‐attempt events per fig.
entry_size	Named numeric vectors controlling entry distribution (the dispersion of the arrival or oviposition‐attempt distribution).
entry_distribution	How per‐species attempt numbers are simulated: Optional “nb” or “lognormal.” See entry_size.
max_entry_table	Maximum number of individuals of each species that can “enter” (arrival or oviposition‐attempt) a fig, scaled by fig size.
entry_priority	(Phase) A list defining which species are active during each temporal phase (1‐A phase, 2‐B phase, or 3‐C phase).
*Reproduction & fecundity*	
fecundity_mean	The mean potential number of eggs per individual wasp.
fecundity_dispersion	The dispersion parameter for the negative binomial distribution modeling individual fecundity.
egg_success_prob	Baseline probability that an arriving or oviposition‐attempting female contributes eggs.
egg_success_prob_by_phase	(Species level) A species‐level list of phase‐specific reproductive‐success probabilities. For each species and developmental phase, the supplied value is used as the probability that an arriving or oviposition‐attempting female contributes eggs during that phase. If no phase‐specific value is supplied, the model uses the baseline egg_success_prob.
*Spatial & trophic interactions*	
layer_preference	A vector specifying the relative preference for ovipositing in core, mid, or outer ovary layers. Species assigned “none” are not given species‐specific layer preferences; their oviposition attempts are allocated according to remaining ovary‐layer availability.
parasitism_prob	In the default layered interaction model, parasitoid reproduction is constrained by defined host availability and ovary‐layer availability rather than by this probability parameter. Used only if use_supplemental_parasitism = TRUE.
interaction_matrix	An empirically‐derived species‐species correlation matrix from a hierarchical Bayesian joint species distribution model (default = NULL). This module can be activated via the interaction_weight parameter to test the effect of including statistical co‐occurrence patterns in the simulation.
interaction_weight	A multiplier scaling the effect of the interaction_matrix. Set to 0 in the baseline configuration modeling to represent a purely mechanistic model.
species_roles	(Species level) A list defining each species' functional guild (pollinator, galler, parasitoid) and host‐parasitoid links.

#### 2.3.1 Initializing in Fig Structure and Floral Resources

Each fig is initialized with a diameter drawn from a truncated normal distribution (default: mean = 2.5 cm; SD = 1.2 cm; bounds = 1.3 to 4.0 cm), based on field data (Zhang et al. 2006; Jauharlina et al. 2017). The number of female flowers (ovules) was derived from empirical fits (diameter ~ *D* ^alpha^, alpha = 1.3) and yields values within the range of 4000 to 11,000, consistent with previous records (Wang et al. 2008, 2014; Ramadhani et al. 2022). These ovules constitute the total floral resource pool available for both seed formation and ovule‐occupying wasp reproduction (i.e., pollinators and gallers).

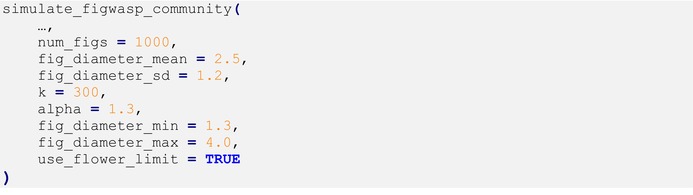

#### 2.3.2 Foundress Abundance and Oviposition Stochasticity

The number of foundresses entering a single fig is empirically known to be highly variable for all interacting species, not just the pollinator. Published studies on *F. racemosa* , for instance, report a wide range for the pollinator (*Ceratosolen* sp.), typically from one to several dozen individuals, with observed means varying substantially across seasons and locations (e.g., 5, 8, or 20). This pattern of high variance and occasional mass‐entry events is a hallmark of the system (Wang, Yang, and Yang 2005; Wang et al. 2014; Emran et al. 2016; Jauharlina et al. 2017). Nonpollinating wasps also exhibit considerable variability in numbers.

To realistically capture this overdispersed, right‐skewed distribution of arrivals, the model assigned each species a stochastic entry process, where the number of entrants into each fig is drawn independently from a lognormal distribution. This distribution was parameterized by two specific values: entry_mu (mean on the log scale) and entry_size (standard deviation on the log scale). The resulting heavy‐tailed entry distributions reproduce both the common low‐entry cases and the rare high‐entry events observed in empirical studies.

For sensitivity analysis or alternative model formulations, the entry process can instead be defined using a negative binomial distribution, also governed by entry_mu and entry_size. This formulation is widely used for modeling overdispersed count data in ecology (Lindén and Mäntyniemi 2011). Unless otherwise noted, all simulations in this study employ the lognormal distribution as the default entry mechanism.

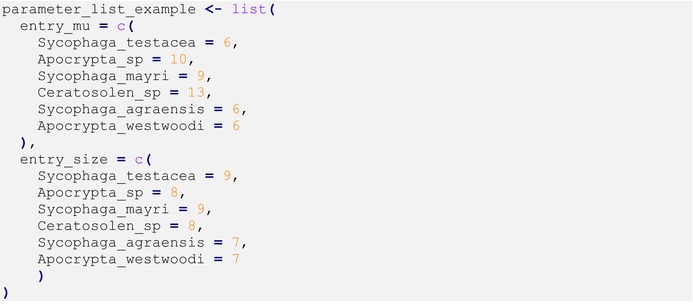

#### 2.3.3 Fig Wasp Emergence and Seed Production

For each species, emergent adults are computed as the number of successfully laid eggs minus those parasitized, assuming all viable eggs develop unless parasitized. Ovules not occupied by wasp eggs are available for seed formation (Emran et al. 2016; Ramadhani et al. 2022), with each ovule having a per‐ovule pollination success probability (default = 0.98). Resource use is tracked per fig to ensure consistency: the total number of ovules must equal the sum of seeds, failed ovules, and eggs.

#### 2.3.4 Simulating Wasp Entry and Priority

The model simulates wasp arrival not as the physical entry of individuals into the fig, but as a stochastic process representing successful oviposition. In this framework, again, an “entrant” is defined as a reproductive female's arrival at a fig or oviposition‐attempt event, an abstraction that captures realized reproductive input. This approach bypasses the need to explicitly model pre‐oviposition factors such as entry failure, host defense, or adult mortality.

Entry phases reflect well‐documented phenology in *F. racemosa* , with pollinators and gallers (i.e., ovule‐occupying wasps) arriving first, followed by parasitoids (Cook and Segar 2010; Cruaud, Jabbour‐Zahab, Genson, Kjellberg, et al. 2011; Aung et al. 2020, 2022).

Fig wasp arrival is temporally structured into three sequential phases governed by the entry_priority parameter. These values can optionally be modified by an interaction matrix when competitive or facilitative effects are activated. To reflect physical constraints imposed by fig size, maximum entry counts (max_entry_table) are scaled according to fig diameter.

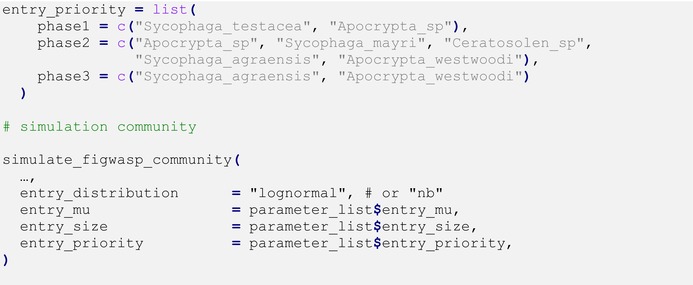

#### 2.3.5 Fecundity and Oviposition Success

Distributions were specified per species, calibrated using observed emergence data. Phase‐specific oviposition success probabilities account for behavioral variation and environmental noise. To simplify the model and focus on oviposition‐based resource allocation, sex ratios were not simulated and no distinction was made between male and female wasps. Each ovipositing individual is treated as a reproductive unit with identical fecundity potential. This assumption is reasonable given that reproductive output in this system is primarily determined by female behavior (females carry eggs and search for new hosts), and male contribution is indirect.

Each wasp is modeled as an individual, with fecundity drawn from a species‐specific distribution. Successful oviposition is probabilistic and phase‐specific (parameterized by egg_success_prob_by_phase). For ovule‐occupying species, total oviposition across all individuals is constrained not to exceed the available ovule pool.

Assigning phase‐specific oviposition success probabilities (0–1) for each species across fig developmental phases, allowing the model to explicitly test temporal heterogeneity, where offspring success depends critically on changing conditions within the fig, such as host phenology or competitor density.

Only ovule‐occupying wasps (pollinators and gallers) directly consume floral space. Parasitic and hyperparasitic species depend on hosts occupying ovules and thus do not consume floral resources themselves. The spatial layering of ovules (core, mid, outer) is based on fig anatomy and represents accessibility constraints based on ovipositor length (Ganeshaiah et al. 1995; Kjellberg et al. 2005). If the use_layering option is enabled, ovules are partitioned into concentric spatial layers and each species has a probabilistic preference influencing oviposition efficiency by layer. For parasitoids, successful oviposition is limited by the number of host eggs available in the same layer, reflecting spatial matching constraints and the commonly reported stratification of hosts, parasitoids, and seeds within figs (Dunn et al. 2008). This pattern is also widely discussed as contributing to mutualism stability by creating enemy‐free space for pollinator offspring. When a species has no species‐specific ovary‐layer preference, its layer_preference value can be set to “none.”

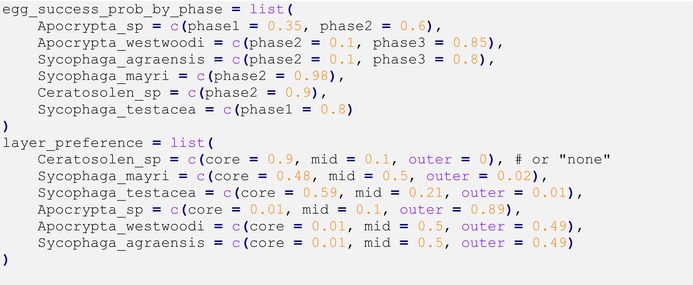

#### 2.3.6 Host‐Dependent Parasitism

In the model configurations, parasitoid reproduction is fundamentally constrained by the presence of their hosts (pollinators or gallers). This core dependency is hard‐coded into the main simulation loop, ensuring ecological realism by preventing parasitoids from ovipositing in the absence of a suitable host. Parasitism occurs only if host eggs are available and a random draw exceeds the species‐specific parasitism_prob threshold. Parasitoid egg numbers are capped by the number of viable host eggs.

The use_supplemental_parasitism parameter (default = FALSE) controls an additional, secondary mechanism. When activated (TRUE), it grants parasitoids that may have failed to oviposit during the primary stochastic entry process a second opportunity to reproduce, triggered probabilistically based on the final host abundance within the fig. This two‐tiered design supports flexible exploration of parasitism dynamics while preserving strict host limitations under all settings. Importantly, disabling this module does not permit biologically implausible outcomes; parasitoids remain incapable of reproducing without hosts.

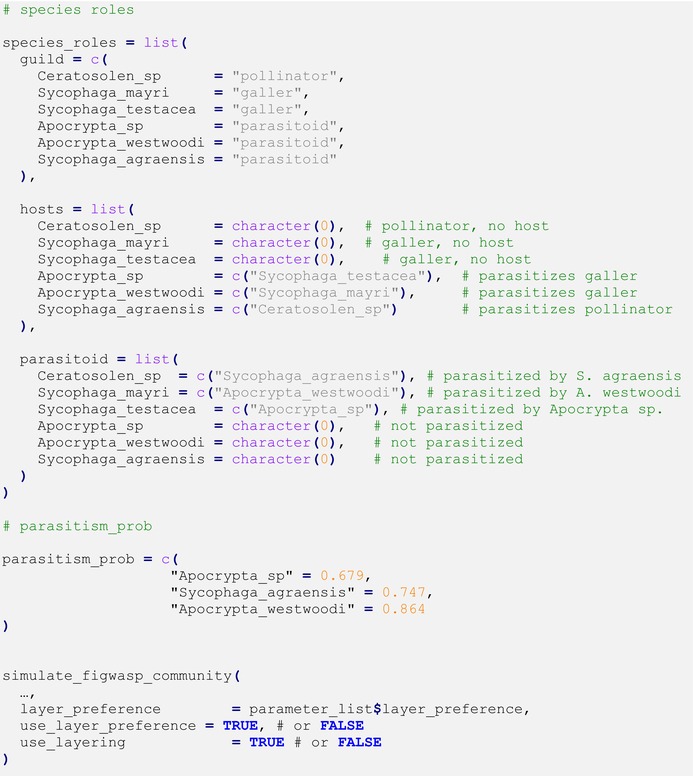

#### 2.3.7 Host Sanctions, Nutrient Sink Strength Hypothesis, and Fig Abortion

Abortion is triggered when ovule usage by wasps exceeds a sanctioning threshold. This threshold is implemented via a logistic function and reflects both theoretical predictions and empirical findings that trees may sanction figs with low pollination or excessive parasitism (Jandér and Herre 2010; Wang et al. 2014).

If enable_drop is activated, each fig has a probability of abortion based on how far the wasp ovule usage exceeds a predefined host_sanction threshold (default = 80% resource consumed). This is implemented as a sigmoid (logistic) function (Yin et al. 2003). When abortion occurs, emergence and seed values are nullified (unless drop_cancels_emergence = FALSE), simulating ecological failure of the fig as a reproductive unit.

The recent nutrient sink strength hypothesis for fig abortion was also adopted in the simulation (Segar et al. 2025) and can be activated (use_sink_strength = TRUE). We quantified sink strength for each galler species, including pollinator and nonpollinator galler, because only galler species directly utilize flowers and other parasitoid wasps do not use flowers. To simplify the model, we assume that sink strength is the same for pollinating and nonpollinating gallers and that the relationship between the number of galler wasps and sink strength is linear (sink_linear_coef, default = 1; this relationship is unlikely to be strictly linear in reality and can be adjusted as needed). For comparison, the sink strength contributions of gallers and seeds were weighted by two parameters to calculate total sink strength within a fig. The default weights for galler sink strength (sink_w_gall) and seed sink strength (sink_w_seed) are set to 1 and 1.5, respectively, following observation reported previously (Wang et al. 2014; Wang, Miao, and Peng 2020). Then, when fig‐level sink strength exceeds the allowable range (reflecting nutrient and energy allocation in a fig), from sink_min_prop (default = 0.2) to sink_max_prop (default = 0.95), the fig is marked as aborted.

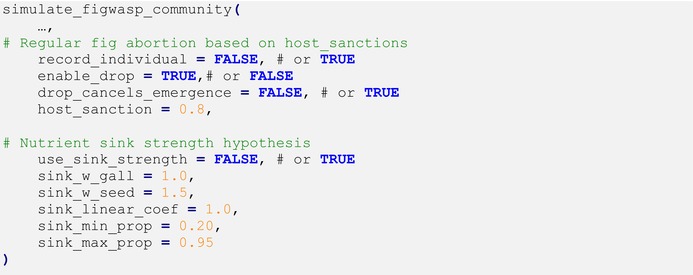

### 2.4 Model Calibration and Parameterization

To parameterize the baseline configuration modeling with ecologically plausible and high‐performing values, we implemented a systematic, two‐stage calibration strategy designed to find a single optimal parameter set that best reproduces the central tendencies of the observed community.

#### 2.4.1 Biologically‐Informed Initialization

Initial parameter values, more than 60 life‐history and interaction parameters, in the model were assigned based on: (1) direct empirical estimates from published studies on *F. racemosa* system; (2) indirect inference from ecologically similar species or functional guilds; (3) established ecological principles such as life‐history trade‐offs; and (4) symmetry assumptions when no species‐specific information was available.

#### 2.4.2 Parameter Space Exploration via Latin Hypercube Sampling

We used high‐resolution empirical data of per‐fig communities (“Observed Data,” *n* = 935 figs) to calibrate parameters and evaluate model performance. In what follows we refer to this as the observed data. Details on sampling, identification, and data curation are described in Aung et al. (2022).

For each parameter, we defined a search range spanning ±30% of its initial value, with biologically sensible hard limits where applicable (e.g., probabilities capped at 1.0). To efficiently explore this high‐dimensional parameter space and search for optimal parameter sets, we employed Latin Hypercube Sampling (LSH) to generate 50,000 unique candidate parameter sets (Helton and Davis 2003). The entire process was implemented using the explore_parameter_space_lhs() function within the figsimR package. The performance of each candidate parameter set was evaluated via a robust loss function, defined as the sum of squared errors between the simulated and observed values. The values compared were the ensemble means of six core community metrics: species richness, Pielou's evenness, mean Bray–Curtis dissimilarity, network connectance, nestedness, and modularity. To account for stochasticity inherent in the assembly process, each simulated ensemble mean was calculated from 200 figs from 500 bootstrap replicates drawn from an underlying simulation of 1000 figs. All evaluations were parallelized across 7 cores to ensure computational feasibility. The loss was calculated as:

$$\text{Loss}=\sum_{i=1}^{n}{\left({\bar{x}}_{i}^{\mathrm{sim}}-{\bar{x}}_{i}^{\mathrm{obs}}\right)}^{2}$$

where ${\bar{x}}_{i}^{\mathrm{sim}}$ and ${\bar{x}}_{i}^{\mathrm{obs}}$ represent the ensemble means of metric from the simulated and observed datasets, respectively. To account for stochasticity in community assembly, each simulated mean was estimated from 500 bootstrap replicates (each consisting of 200 figs), drawn from a pool of 1000 simulated figs per parameter set. This computationally intensive procedure was implemented in figsimR package and parallelized across seven cores. The parameter set with the lowest loss value was selected as the final configuration of the baseline configuration modeling.

Upon completion of the search, the parameter set that yielded the minimum loss score was designated as the optimal parameterization. Initial estimates and final optimized values for the baseline configuration modeling (BCM) parameters were provided (Table S2). This single parameter set was then fixed and used to define the BCM in all subsequent analyses, including the mechanism‐perturbation experiments and the final validation against the full distribution of observed data.

### 2.5 Descriptions of Input Data and Simulated Output

#### 2.5.1 Data Preparing and Inputs

A parameter list (e.g., parameter_list_default) is used together with role tables that define species, guilds, trophic links, and optional layer preferences. This is a long, information‐rich list. Users also can prepare their own specific parameter list by following the example list. The list names for per element should be the same; otherwise, functions in the package can return errors later. Simply put, the key requirement is to build a list with the same structure and then modify values according to your needs. The interaction_matrix can be empty, because it is typically derived from empirical data or observed data but can be reconstructed later. These values can be approximate or assessed estimates, and they can be calibrated against the observed data in users' studies.

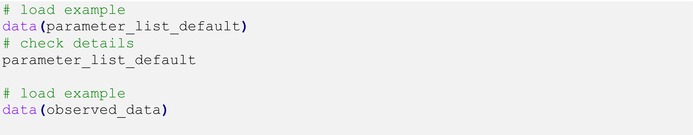

The observed_data includes the community data from the literature, including fig wasp emergence counts per fig for all observed species: row being each fig and column being the fig wasp species, filled by the individual counts. The species name in observed_data and parameter list must be the same. There is an example, showing how to prepare the input list by following the list structure above:

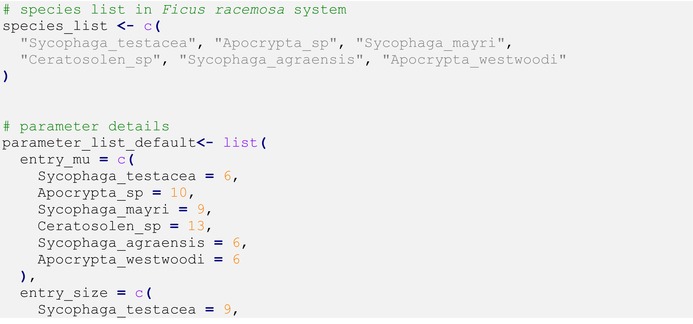

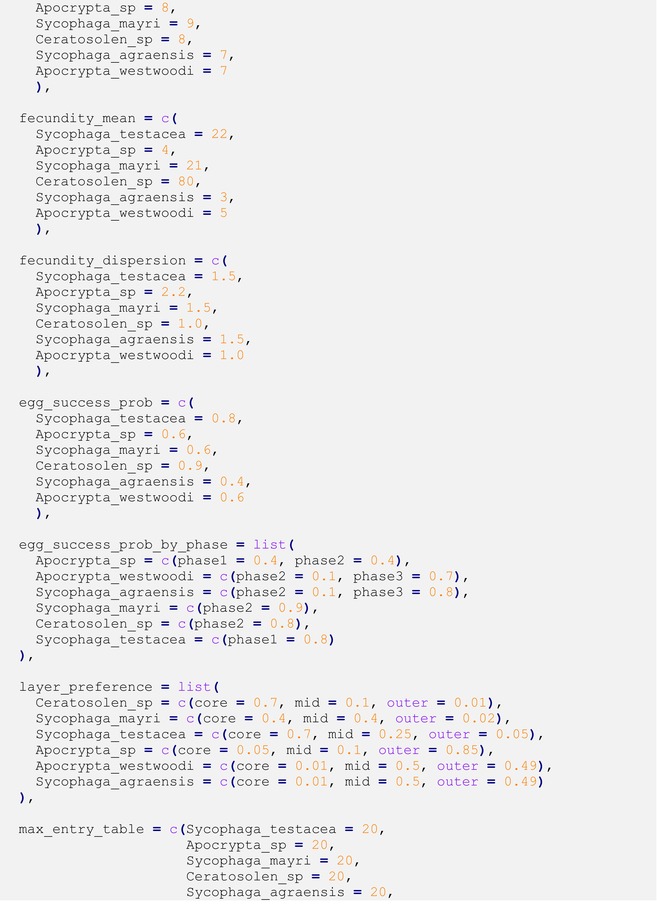

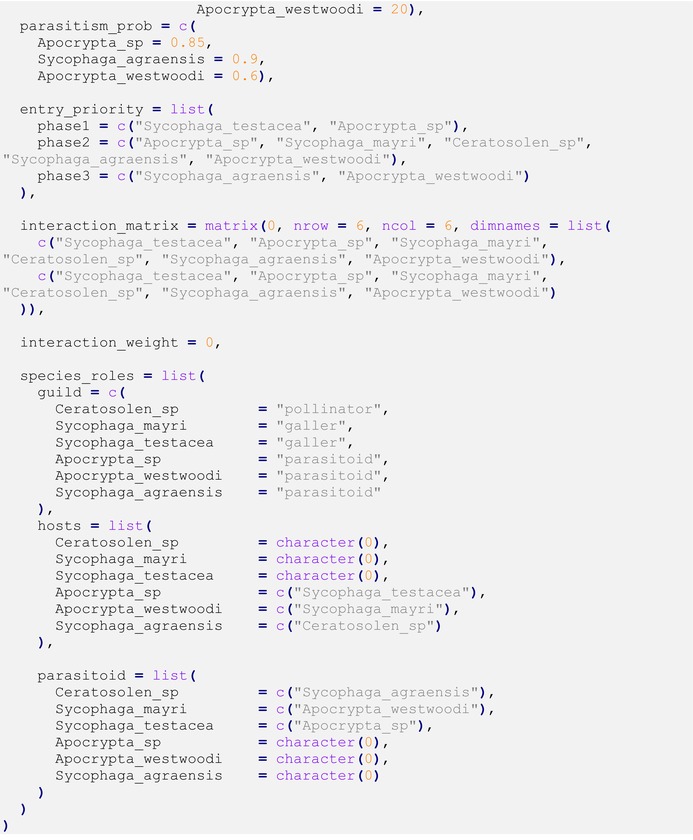

#### 2.5.2 Parameter Calibrations

The first step is to remove the interaction matrix if it exists (it can be estimated from the observed dataset via running the HMSC model) and convert the parameter list into the recognized format for LHS optimization, setting the percentage range around each parameter's base value to use for lower and upper bounds (default = 0.3). The second step is to calculate the metrics from the observed data, allowing the LHS to find the minimum loss for each metric. The LHS optimization can take a long time, depending on the parameter combinations to test and the provided available CPU number. Then the optimized parameter results are needed to be reformatted back to the original structure.

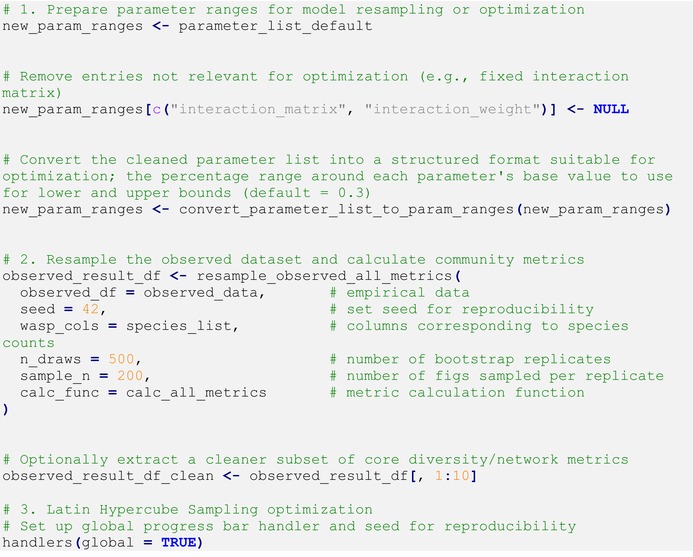

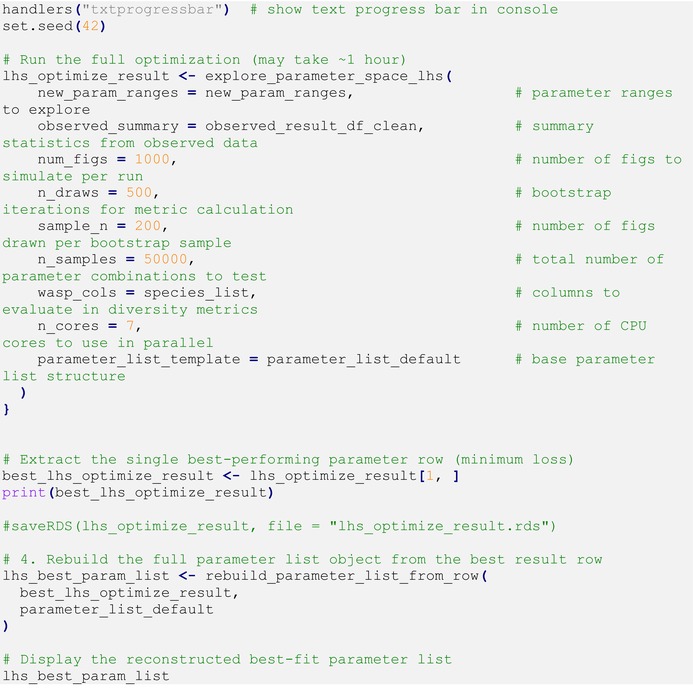

#### 2.5.3 Simulation and Output

The output list includes a summary data frame with per‐fig simulation outputs, such as fig diameter, flower count, entry species, eggs per species, emergence individual per species, seeds, and fig fate (abortion or not) and a list of individual‐level egg counts (optional record_individual = TRUE).

Core invariants enforced and tested: (1) resource closure per fig, that is, eggs + seeds + failed ovules = total ovules; (2) parasitoid/hyperparasitoid eggs never exceed host eggs; (3) entries, eggs, and emergences are nonnegative integers.

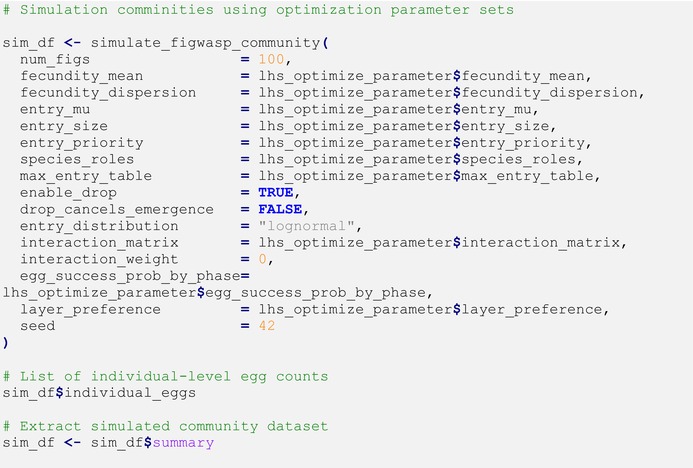

### 2.6 Advanced Feature: The “Knockout” Framework for Mechanism Assessment

Switch mechanisms on or off, *figsimR* allows users to disable or perturb any module to quantify its marginal contribution to community structure and variance coverage. This workflow can be easily scripted to generate matched baseline vs. “knockout” runs and can be combined with parallel tools (e.g., furrr (Vaughan and Dancho 2022)), thereby turning simulation outputs into computational experiments.

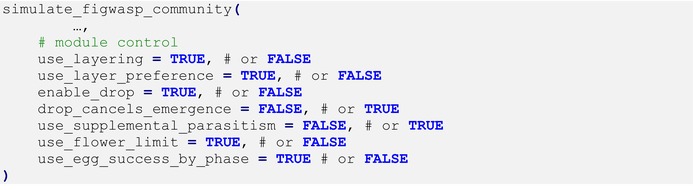

## 3 Results

### 3.1 Parameter Setup and Running a Simulation

The simulation was run with the default parameter list. The optimized parameter sets were used to simulate the baseline configuration modeling (BCM) in following analyses. The descriptions and calibrations of parameters were explained above. Here we only focused on the simulation workflow.

### 3.2 Analyzing Simulation Outputs and Assessing Goodness‐of‐Fit

**FIGURE 2:**
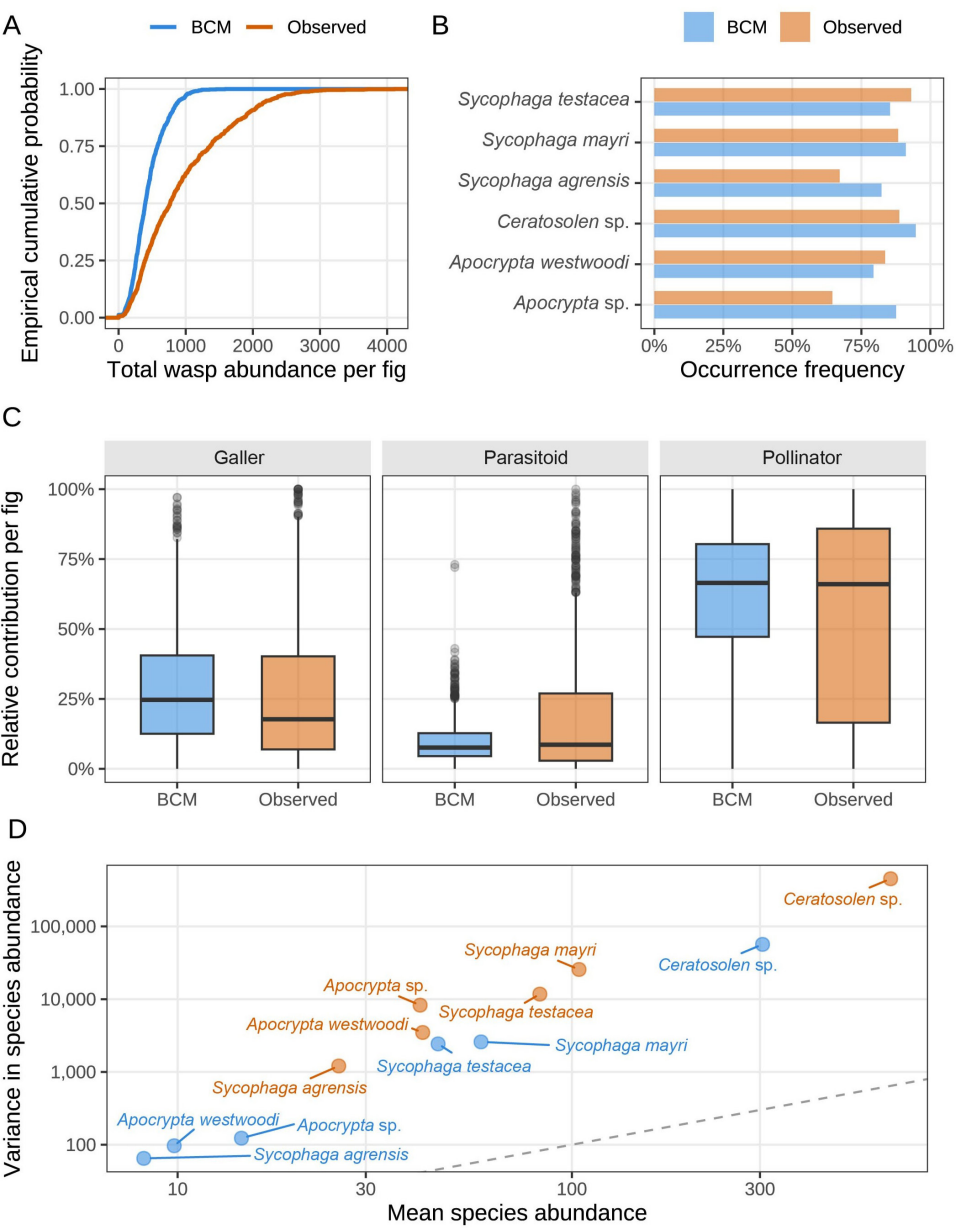
Primary diagnostic comparison between observed and simulated fig‐wasp communities. (A) Empirical cumulative distribution of total wasp abundance per fig. (B) Species occurrence frequency across figs. (C) Relative guild contribution per fig for gallers, parasitoids, and pollinators. (D) Mean–variance relationship of species abundance across figs.

**FIGURE 3:**
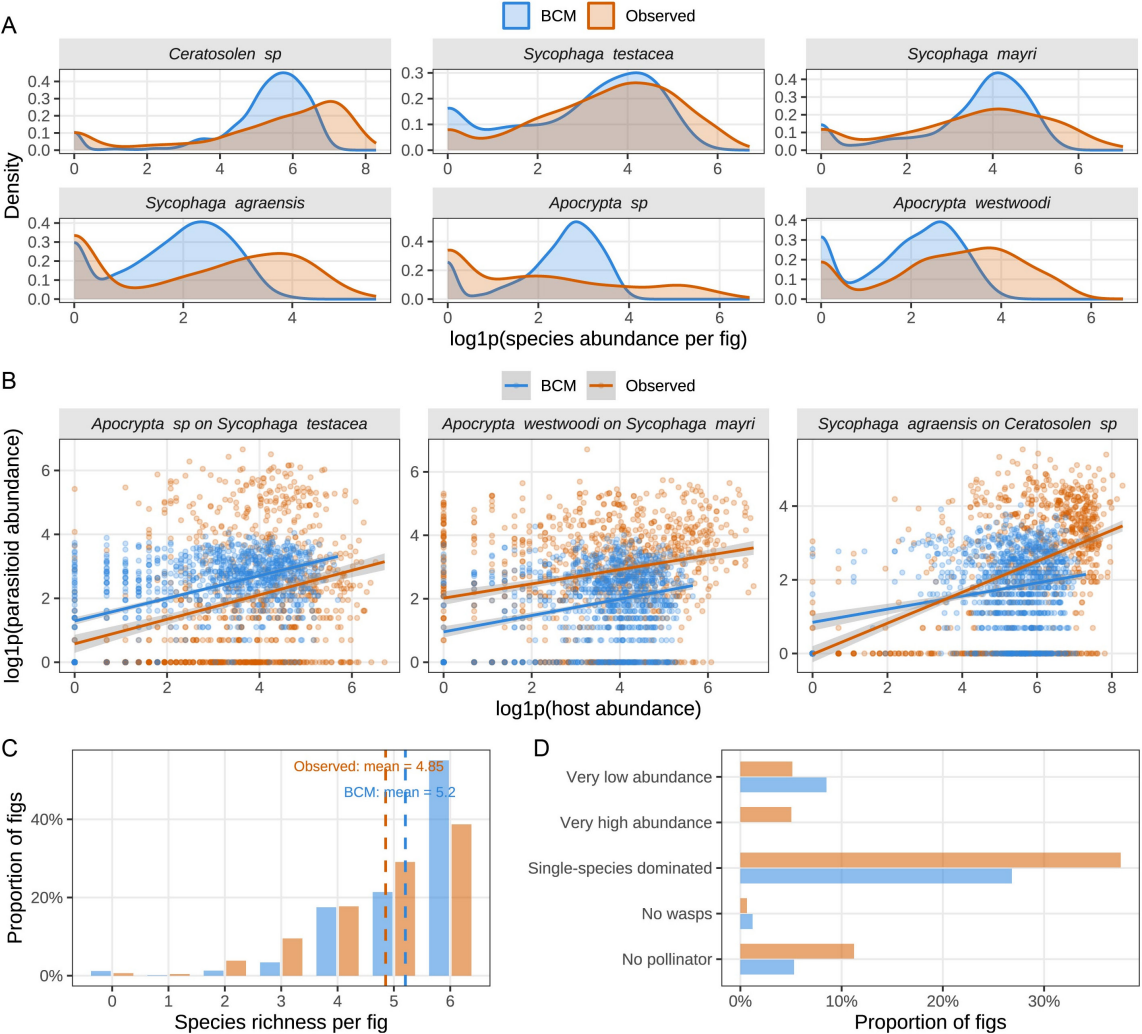
Species‐level and extreme‐state comparison of observed and BCM‐simulated fig‐wasp communities. (A) species‐specific abundance density distributions; (B) host–parasitoid conditional abundance relationships; (C) species richness per fig; (D) frequencies of extreme community states.

Using the calibrated parameters, we generated a baseline pool of 1000 simulated figs. Then, we evaluated the simulated communities against the observed fig‐level community. At the community level, the simulated data and observed data both had broadly the same occurrence frequencies and guild‐level composition, but the simulated data generated a narrower distribution of total wasp abundance and lower species‐level abundance variance than the observed data (Figure 2). At the species level, species abundance densities and host–parasitoid relationships further supported that the observed data showed a wider range of abundance states than the simulated data (Figure 3). The simulated communities had slightly higher mean richness per fig, whereas the observed data contained a greater proportion of extreme community states, especially single‐species dominated figs and figs without pollinators. These results suggest that the developed baseline model in this study approximates some mean community properties but does not fully reproduce the observed among‐fig variability.

In addition, we also evaluated distribution features of communities between simulated and observed data, rather than relying only on single community summaries. To achieve this, for each of 500 bootstrap replicates, we sampled 200 figs from this pool and computed richness, Pielou's evenness, distance to the multivariate centroid, and pairwise Bray–Curtis dissimilarity for both the observed data and the BCM simulations.

**FIGURE 4:**
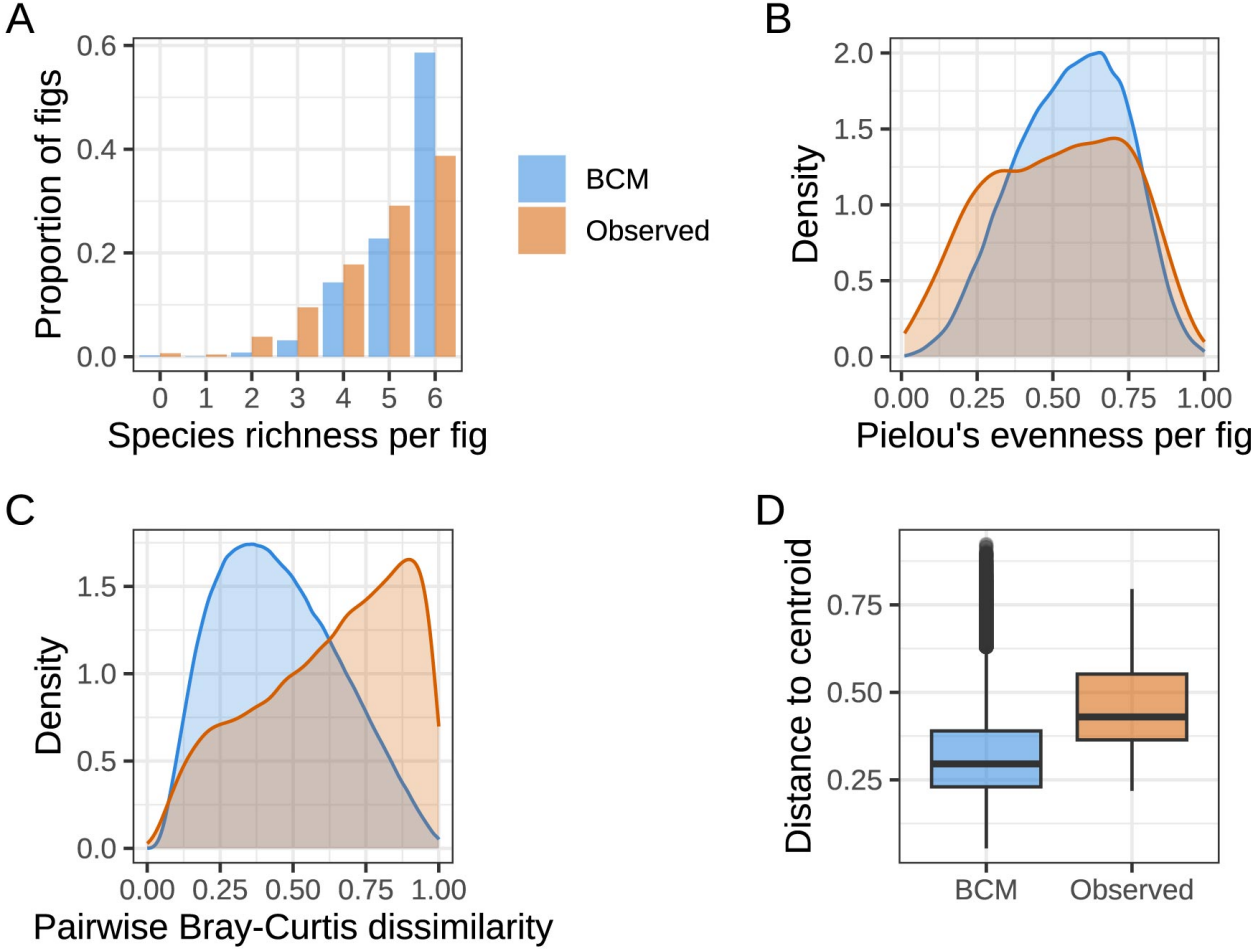
Bootstrap comparison of observed and BCM community distributions. BCM simulations generated higher richness and evenness but lower compositional dissimilarity and dispersion. Each comparison was based on bootstrap resamples (500 × 200 from a pool of 1000 simulated figs).

The results showed that BCM simulated data produced more high richness figs and higher evenness, but lower Bray–Curtis dissimilarity and lower multivariate dispersion than observed data (Figure 4, Figures S2 and S3). When observed metric means were standardized relative to the BCM bootstrap distributions, all six metrics fell outside the BCM 95% predictive intervals (Figure S4).

Overall, these results showed that although the BCM reproduced some aspects of mean community structure, it generated communities that were less compositionally heterogeneous among figs. The pattern—systematic underestimation of community heterogeneity and unevenness, alongside occasional matching for some metrics mean value—points to missing, nonrandom drivers (e.g., host heterogeneity and disturbance from predators) that reshape the full distributions of community properties.

### 3.3 The “Knockout” Framework for Mechanism Assessment

We evaluated each intrinsic module along two complementary axes: (1) mean fit, quantified as the increase in total loss relative to the calibrated BCM ($\text{ΔLoss}={\text{Loss}}_{\mathrm{KO}}-{\text{Loss}}_{\mathrm{BCM}}$; SSE over the six mean metrics), and (2) variance adequacy, quantified as the change in predictive coverage of observed quantiles ($\text{ΔCoverage}={\text{Coverage}}_{\mathrm{KO}}-{\text{Coverage}}_{\mathrm{BCM}}$, across six metrics × five quantiles) together with the percent change in multivariate dispersion (Δρ) from betadisper in vegan package (Oksanen et al. 2025).

We altered one module at a time—spatial partitioning, layer preference, phase‐specific success, priority effects, host sanction, and sink strength—while holding all other parameters at the BCM values (values in the baseline configuration model), so that any change in mean fit or variance coverage can be attributed to the focal mechanism rather than re‐tuning the parameter set. Each scenario followed the simulation workflow (1000 figs per run; bootstrap 500 × 200) and was summarized by ΔLoss and Δρ.

**FIGURE 5:**
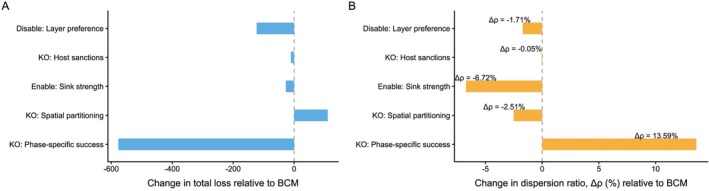
Using mechanism toggles to compare scenarios. Each bar summarizes how a mechanism‐off scenario differs from a baseline configuration modeling (BCM) when both are simulated and analyzed with the same pipeline. (A) Mean fit. Horizontal bars show $\text{ΔLoss}={\text{Loss}}_{\mathrm{KO}}-{\text{Loss}}_{\mathrm{BCM}}$; bars extending to the right indicate worse mean fit than the BCM. (B) Variance adequacy. Bars show the normalized out‐of‐band gap, which in turn aggregates how far the observed quantiles lie outside the model's 95% predictive intervals in units of half‐width (larger values = poorer variance coverage). Text labels report the concurrent change in multivariate dispersion (Δρ), computed as the percent change in the ratio of mean distances to group centroids (Observed/Model) relative to the BCM.

**FIGURE 6:**
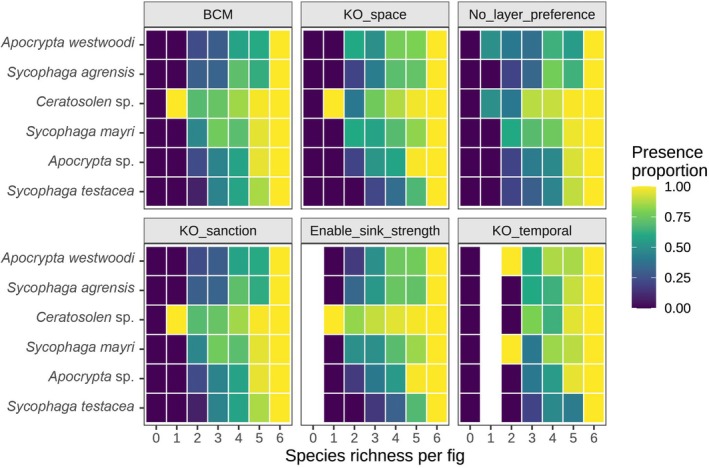
The heatmap of species occurrence across fig‐level richness gradients under baseline (BCM) and mechanism‐altered simulations. Rows represent each fig wasp species; columns represent species richness per fig. Colors indicate the proportion of figs within each richness class in which each species was present.

The knockout simulations uncovered strongly asymmetric contributions of individual mechanisms, with effects that differ in both magnitude and direction across mean fit and variance coverage. In particular, mechanisms that improve mean fit do not necessarily improve variance adequacy, and vice versa, highlighting a clear trade‐off between mechanisms in the communities (Figure 5 and Figure S5). Strong trade‐offs between mean fit and variance adequacy across model components were observed. Among the tested five modules, the phase‐specific success (use_egg_success_by_phase = FALSE) module produced the strongest effect on communities. Disabling it substantially reduced total loss relative to the BCM (ΔLoss = −576), and the dispersion ratio increased markedly relative to the BCM (Δρ = +13.59%; Figure 5). Spatial partitioning showed the opposite pattern (ΔLoss = 110, Δρ = −2.51%). This result indicates that the effect of spatial structure is configuration‐dependent. The module affects community composition, but its removal does not necessarily increase the mismatch in among‐fig dispersion under the current BCM parameterization (Figures 5 and 6).

Host sanctions and sink‐strength filtering had comparatively weaker or more targeted effects. Disabling host sanctions produced little change in either mean fit or dispersion ratio (ΔLoss = −10.4, Δρ = −0.05%). Compared with disabling host sanctions, enabling the sink‐strength rule reduced the dispersion ratio more strongly (Δρ = −6.72%) with a small change in total loss (ΔLoss = −27). Disabling species‐specific layer preference improved mean fit but caused only a small reduction in the dispersion ratio (ΔLoss = −123, Δρ = −1.71%).

## 4 Discussion

We introduce *figsimR*, a modular R package that integrates a mechanism‐explicit, per‐fig agent‐based simulator. To our knowledge, it is the first simulation model parameterized with fig‐level *Ficus racemosa* community data. Beyond providing a simulation tool, figsimR also offers a framework for asking which observed community properties can be generated from known, empirically grounded biological mechanisms and which properties remain insufficiently explained. This package makes three primary contributions: (1) formalizing known biological information in this system—such as species roles, trophic links, entry rules, oviposition windows, and flower resource constraints—into structured, calibratable model inputs; (2) implementing a reproducible simulation–metrics–comparison workflow that allows users to integrate both mean fit and variance coverage as joint diagnostic criteria for assessing mechanistic effects on community assembly; and (3) introducing “knockout” designs to quantify the effects of a single mechanism, or interactions among multiple mechanisms, on community assembly.

Overall, simulated communities, generated by *figsimR*, approximated selected broad mean‐level features, but spread and tails remain too small, especially for evenness and β‐diversity. The simulated communities consistently showed lower variation than the observed communities (Figures 2 and 3). This pattern indicates that the known baseline configuration captures the central tendency of some properties, such as occurrence frequencies and broad species composition. This mismatch does not imply that the simulation fails. Instead, it likely reflects missing processes not yet included in *figsimR*, or real‐system variation that exceeds what the current parameterization can produce. This diagnostic outcome pinpoints which intrinsic mechanisms approximate selected observed features and where additional drivers are needed. In other words, fig wasp communities are more complex than expected. Our framework therefore dissects mechanisms along mean fit and variance coverage, rather than only mapping the limits of intrinsic mechanisms.

The “knockout” design is a core feature of the *figsimR* package. It allows users to intervene in the model structure, and test and understand the relationship between ecological mechanisms and community assembly as computational experiments. Except for variance inadequacy recorded in simulated communities, the “knockout” framework reveals asymmetric contributions of individual mechanisms and mechanism‐specific mean–variance trade‐off to fig wasp communities. For instance, the differential use of ovary layers, oviposition sites, and temporal niches within figs is the dominant mechanism controlling the variance of fig wasp communities (Ganeshaiah et al. 1995; Kerdelhué et al. 2000; Ghara et al. 2011); this is ecologically intuitive, because fig wasps are primarily separated by spatial layers and host accessibility within figs.

Our simulations were consistent with this convention, the modules most closely related to spatiotemporal niche partitioning, including phase‐specific success, implemented spatial partitioning, and species‐specific layer preferences, contributed strongly to variance structure in the current model configuration. Among the knockouts, phase‐specific success produced the strongest mean–variance trade‐off. These results suggest that a mechanism can be essential for realistic variance while still harming mean fit (Gelman et al. 1996; Evans et al. 2013). Such trade‐offs argue for more context‐dependent interpretations and highlight the value of diagnosing adequacy along two axes—mean fit and variance adequacy—a central challenge in confronting models with data (Gelman and Hill 2006; Melbourne and Hastings 2008; Evans et al. 2013). As a summary, a better mean fit does not guarantee adequate variance; this trade‐off can mislead inference about mechanism effects in ecological studies.

Using this framework to measure and compare the explanatory boundaries of individual mechanisms across systems may provide a new route to understanding insect community assembly in nature. In particular, by quantifying the same mechanism's contribution across systems using comparable diagnostics, this framework allows us to link local interactions to cross‐scale diversity patterns, even when data structures differ. More broadly, comparing how the same mechanism contributes to variations across different systems could help address the foundational challenge of explaining biodiversity patterns across scales (Levin 1992; Gonzalez et al. 2020; Swan et al. 2021).

The *figsimR* package, parameterized using fig wasp communities associated with *F. racemosa* , is also extended to other *Ficus*–fig wasp systems. To fulfill this, users need to customize the inputs dataset following the guidelines and parameter templates presented in the packages. The current implementation also supports communities with multiple pollinating fig wasp species associated with the same *Ficus* species, further increasing its applicability across fig–fig wasp systems. The number of simulated species is also not hard‐coded, and users can supply custom species lists and interaction structures.

The main contribution of the package is a general workflow: biological information on species interactions can calibrate an intrinsic model for mean patterns, and the agent‐based model framework allows us to isolate the focal mechanism effects on community assembly by enabling or disabling a single module. Conceptually, this workflow can be transferred and applied to other systems by replacing species roles and trophic links, and by reconfiguring modules (e.g., light competition and herbivory in grasslands; metabolic antagonism and phage predation in microbiomes). This aim aligns with evaluating how far intrinsic theory goes and quantifying what remains unexplained, echoing pattern‐oriented modeling (Grimm et al. 2005).

However, transferring this workflow to other systems remains challenging because relationships among agents can be highly elaborate and often require long‐term data accumulation (sometimes decades). In addition, fig–fig wasp systems have a distinctive feature: offspring must complete development inside figs, which in turn enables researchers to measure and document species interactions in a relatively stable and consistent environment. Even more important is replicated sampling. Many fig‐level communities can be recorded at the same time because large numbers of figs mature and can be collected within a single harvest period. These properties also highlight why applying the same workflow to many other systems may be less straightforward. That said, other similar enclosed systems, such as Asteraceae flower heads (Fonseca et al. 2005) or globeflowers (Jaeger and Després 1998), may offer comparative units with well‐documented and replicated species interactions.

This figsimR package provides a baseline tool for process‐based simulation of fig wasp communities. In this system, spatiotemporal niche partitioning appears crucial, but the contribution of each implemented module is configuration‐dependent; therefore, measurements of niche‐depth distributions, ovule‐layer accessibility, and ovipositor‐length–layer matching should be among the most efficient ways to reduce uncertainty (Ganeshaiah et al. 1995; Kjellberg et al. 2005). At the same time, the remaining variation points to structured “extrinsic” drivers (e.g., processes outside of the fig and beyond the intrinsic fig–fig wasp interaction rules) that are not yet encoded in the model. Integrating this framework to test whether the relative importance of spatial stratification differs across closely related fig species with contrasting breeding systems (e.g., monoecious and dioecious species) can contribute to understanding how strongly spatial stratification shapes variation in fig wasp communities cross species. The framework can also be improved by integrating extension modules, such as micro‐environmental heterogeneity (per‐fig variability in resources or temperature), colonization stochasticity arising from external spatial context (e.g., predators), and fig variation among trees or seasons (including the spatial distribution of the trees themselves).

Compared to observed communities, the modeling produced less among‐fig variation; one potential reason is that the simulation was generated from a single calibrated parameter set, whereas the observed communities are unlikely to arise from a homogeneous set of biological conditions. As we discussed above, in nature, figs may differ among fig trees, seasons, microclimates, local fig wasp diversity, predator disturbance, and other external contexts that are not yet explicitly integrated in the current model. The reduced variance in the simulation may be interpreted not only as model inadequacy, but also as evidence that real fig‐wasp communities integrate additional layers of environmental and demographic heterogeneity. To simulate the variations of fig wasp communities in natural settings, we recommend that users explore these missing variation sources by perturbing modeling and running multiple simulations, such as parameter changes, repeated simulations with enable/disable modules, or defining alternative parameter sets that represent different ecological contexts. Such perturbing simulations can also help evaluate which unmodeled external factors are most likely to expand the simulated distribution toward the observed communities.

## Author Contributions

**Yiyi Dong:** conceptualization (lead), formal analysis (lead), software (lead), visualization (lead), writing – original draft (lead), writing – review and editing (equal). **Simon T. Segar:** methodology (equal), writing – review and editing (equal). **Qingbei Weng:** conceptualization (supporting), funding acquisition (supporting), writing – original draft (equal), writing – review and editing (equal).

## Funding

This research was supported by grants from the Joint Fund of the National Natural Science Foundation of China and the Karst Science Research Center of Guizhou Province (U1812401) and the Provincial Program on Platform and Talent Development of the Department of Science and Technology of Guizhou, China ([2019] 5617; [2019] 5655).

## Conflicts of Interest

The authors declare no conflicts of interest.

## Supporting information


**Figure S1:** Mechanistic and Temporal Structure of the figsimR Model. (a) Trophic network depicting the six simulated fig wasp species. Nodes were colored by functional guild (pollinator, galler, or parasitoid), and directed edges indicated parasitism from host to parasitoid. (b) Temporal assembly timeline showing the oviposition window of each species across three discrete phases (A: Early, B: Mid, C: Late). Colored bars represented periods of activity, while black arrows indicated host‐dependency—parasitoids can only become active following the entry of their respective hosts.

**Figure S2:** Mean values of four metrics for observed and simulated communities.

**Figure S3:** Visualizing comparisons between simulated (Baseline Configuration Modeling, BCM) and observed data for community metrics. All analyses are based on Hellinger‐transformed species‐by‐fig matrices and Euclidean geometry. (A) Multivariate separation (PCoA). Ellipses are 95% data ellipses (not confidence regions). (B) Multivariate dispersion. Distributions of distances to group centroids (kernel densities) indicate greater spread for the observed data than the BCM. Dashed vertical lines mark group means. (C) Scalar coverage and quantile diagnostics. For six core metrics, shaded bands show the BCM's 95% predictive interval for sample quantiles (bootstrap ensembles of simulated figs; e.g., 500 replicates × 200 figs drawn from a pool of 1000). Orange lines and points are the corresponding observed quantiles (e.g., 5%, 25%, 50%, 75%, 95%). Points outside the band diagnose coverage deficits and tail‐biased departures, mapping the variations for individual properties.

**Figure S4:** Visualizing simulated‐observed comparisons for community metrics. For six metrics, blue intervals show predictive intervals for the baseline configuration modeling (BCM) simulated means estimated from bootstrap resamples (500 × 200 from a pool of 1000 simulated figs). Black points mark the observed means computed with the same pipeline.

**Figure S5:** Distributions of species richness per fig across mechanism‐altered simulations.

**Table S1:** Full parameter list and their descriptions in the figsimR package.

**Table S2:** Initial estimates and final optimized parameters in the Baseline Configuration Modeling (BCM). Initial values were informed by literature and biological reasoning. Final values reflected results from the Latin Hypercube Sampling optimization procedure and were used in all subsequent simulations in the BCM.

## Data Availability

All code and data associated with this study are available in the paper, its Supporting Information, and figsimR package (https://github.com/dongyiyi/figsimR; https://doi.org/10.6084/m9.figshare.29974348).
