# Supplementary figures and images for: figsimR: An R Package for Simulating Fig–Wasp Community Dynamics
Source: Ecol Evol. 2026 Jul 20;16(7):e74018. doi: 10.1002/ece3.74018 (PMC13385217; doi:10.1002/ece3.74018)

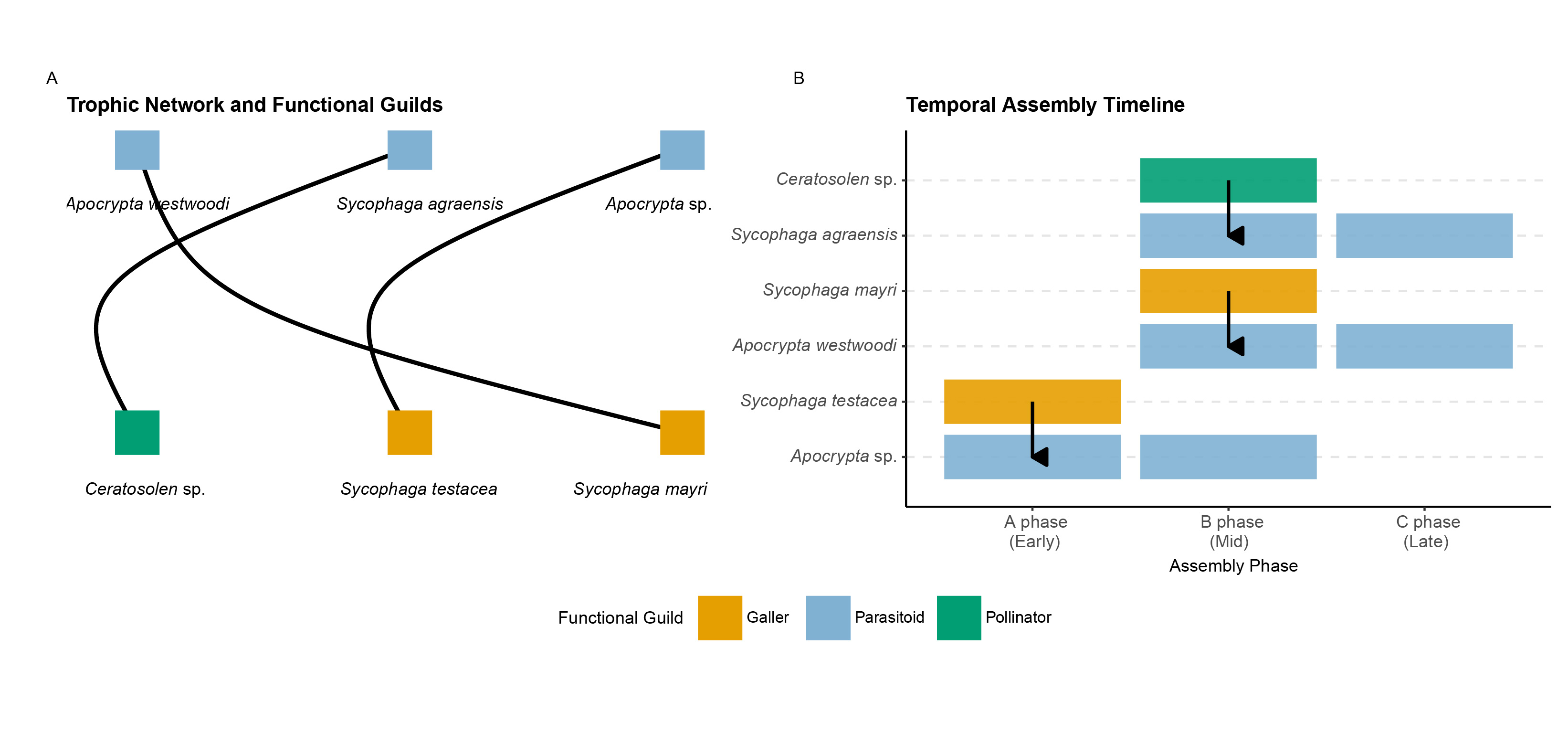

Supplement: Supplementary file 1 — Figure S1: Mechanistic and Temporal Structure of the figsimR Model. (a) Trophic network depicting the six simulated fig wasp species. Nodes were colored by functional guild (pollinator, galler, or parasitoid), and directed edges indicated parasitism from host to parasitoid. (b) Temporal assembly timeline showing the oviposition window of each species across three discrete phases (A: Early, B: Mid, C: Late). Colored bars represented periods of activity, while black arrows indicated host‐dependency—parasitoids can only become active following the entry of their respective hosts. [file ECE3-16-e74018-s001.jpg]

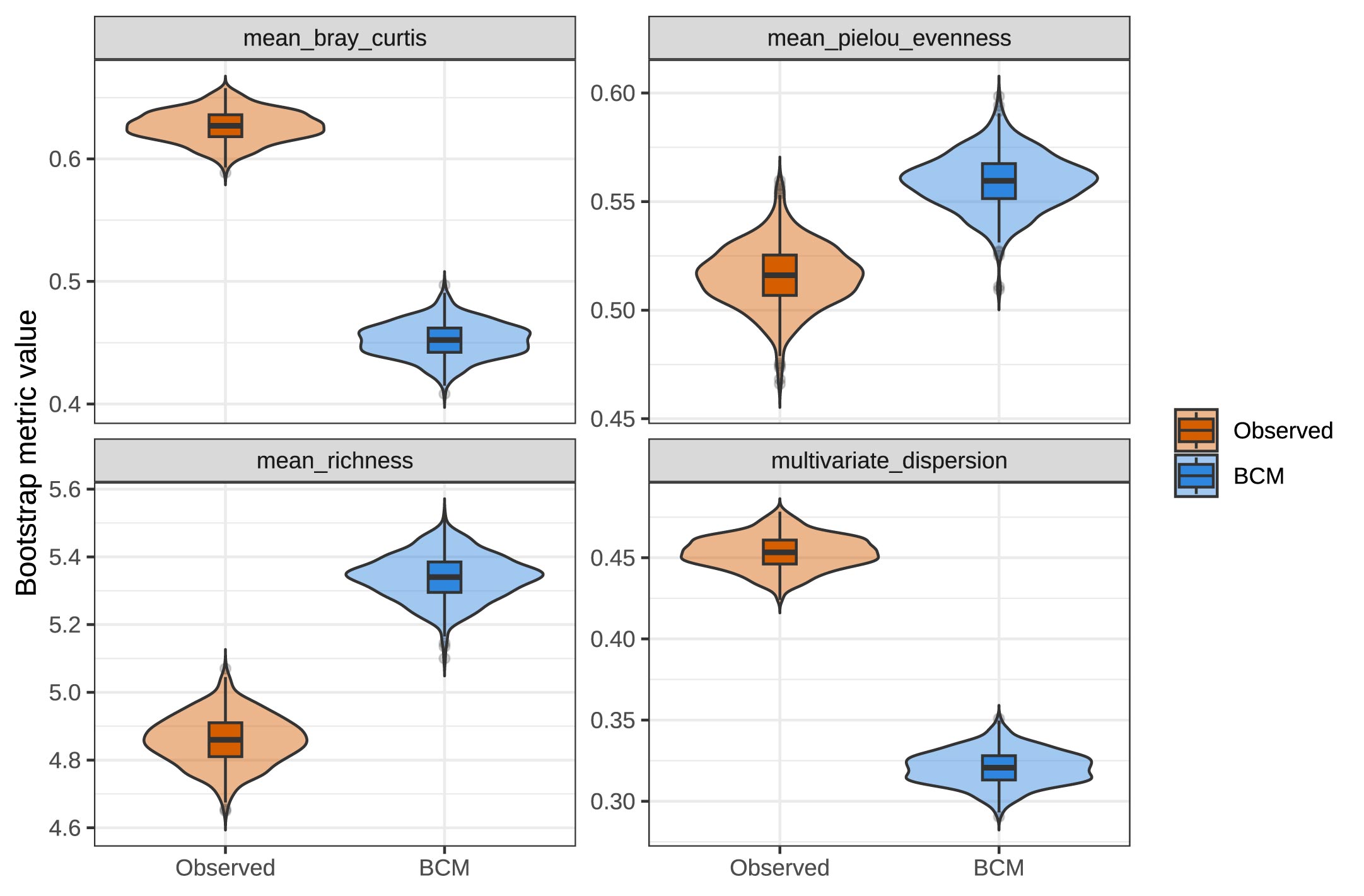

Supplement: Supplementary file 2 — Figure S2: Mean values of four metrics for observed and simulated communities. [file ECE3-16-e74018-s007.jpg]

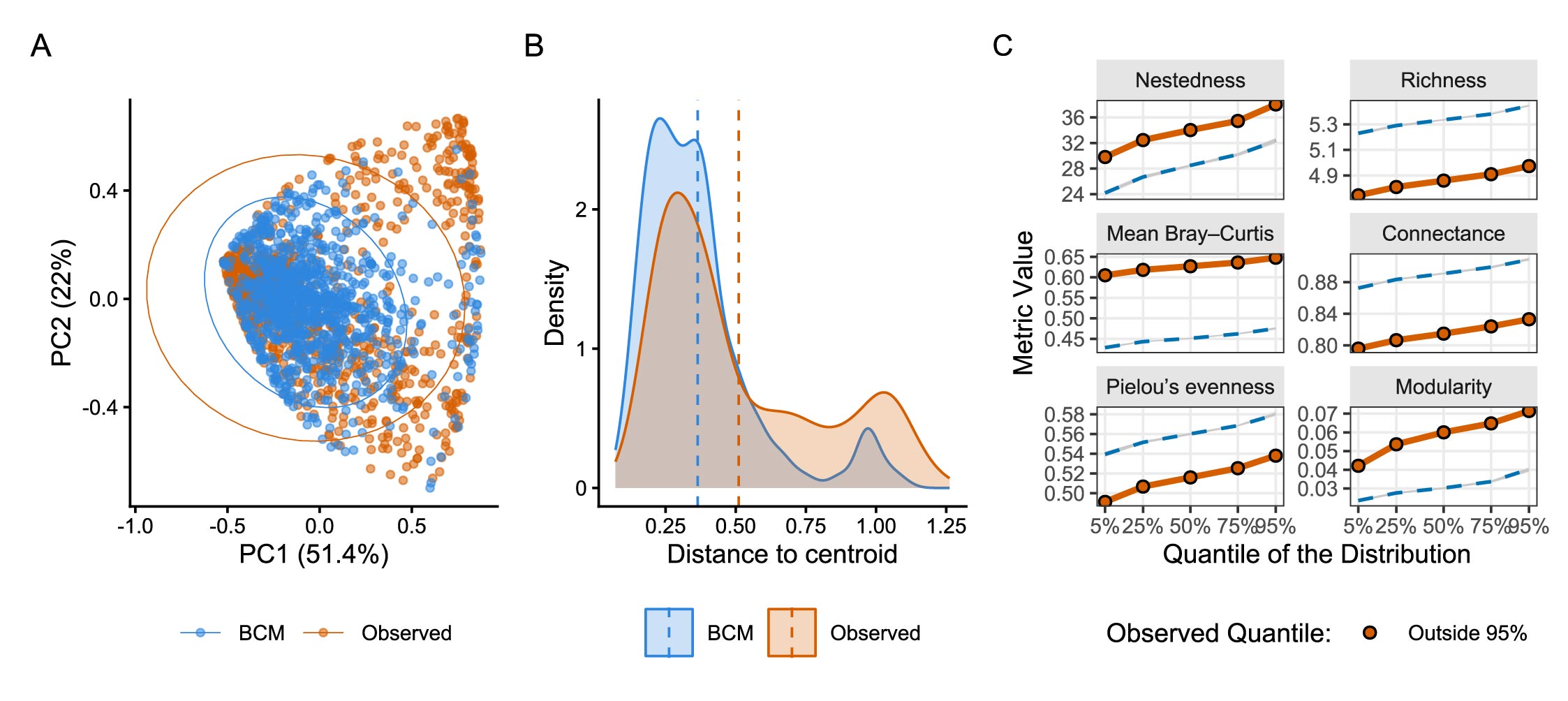

Supplement: Supplementary file 3 — Figure S3: Visualizing comparisons between simulated (Baseline Configuration Modeling, BCM) and observed data for community metrics. All analyses are based on Hellinger‐transformed species‐by‐fig matrices and Euclidean geometry. (A) Multivariate separation (PCoA). Ellipses are 95% data ellipses (not confidence regions). (B) Multivariate dispersion. Distributions of distances to group centroids (kernel densities) indicate greater spread for the observed data than the BCM. Dashed vertical lines mark group means. (C) Scalar coverage and quantile diagnostics. For six core metrics, shaded bands show the BCM's 95% predictive interval for sample quantiles (bootstrap ensembles of simulated figs; e.g., 500 replicates × 200 figs drawn from a pool of 1000). Orange lines and points are the corresponding observed quantiles (e.g., 5%, 25%, 50%, 75%, 95%). Points outside the band diagnose coverage deficits and tail‐biased departures, mapping the variations for individual properties. [file ECE3-16-e74018-s003.jpg]

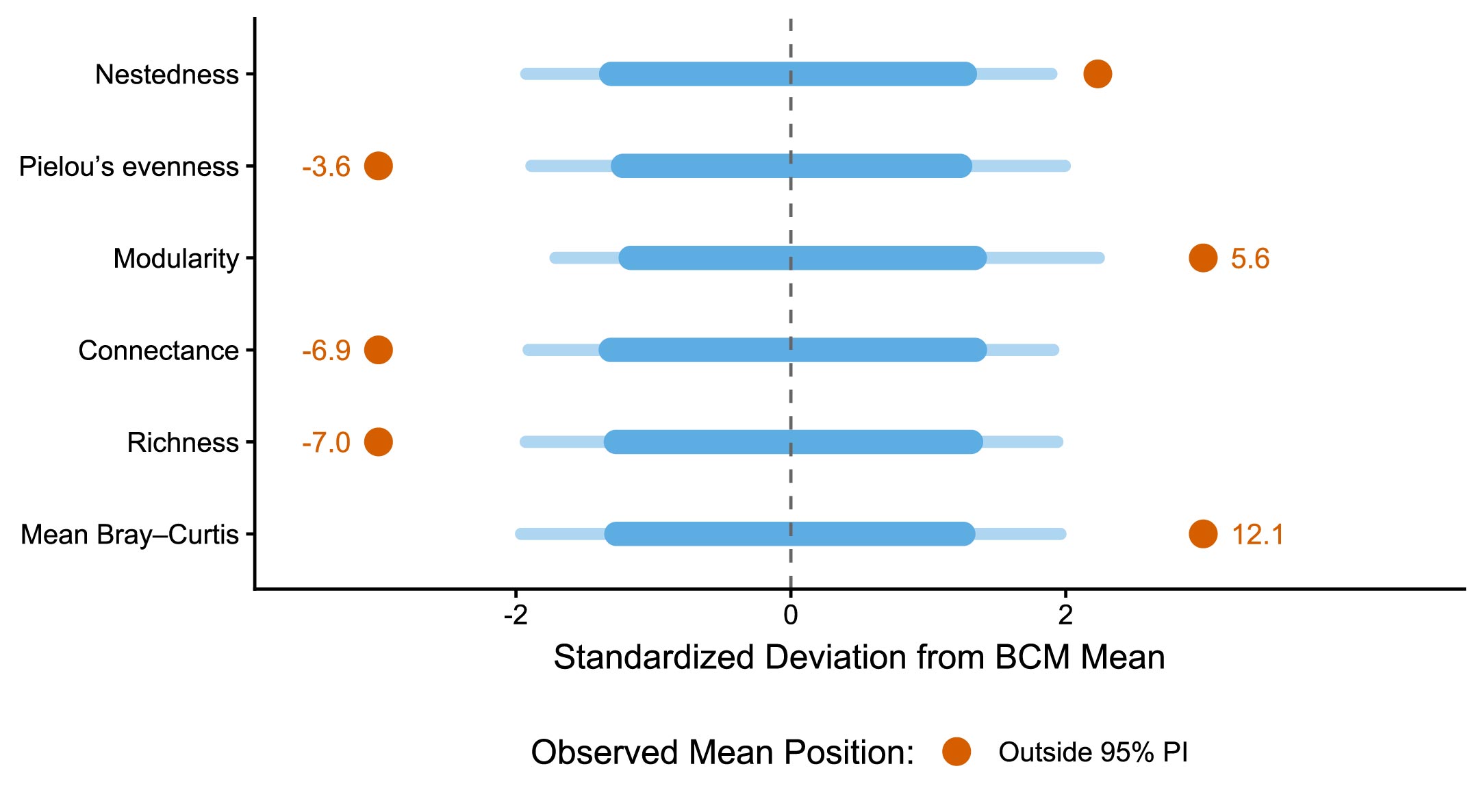

Supplement: Supplementary file 4 — Figure S4: Visualizing simulated‐observed comparisons for community metrics. For six metrics, blue intervals show predictive intervals for the baseline configuration modeling (BCM) simulated means estimated from bootstrap resamples (500 × 200 from a pool of 1000 simulated figs). Black points mark the observed means computed with the same pipeline. [file ECE3-16-e74018-s002.jpg]

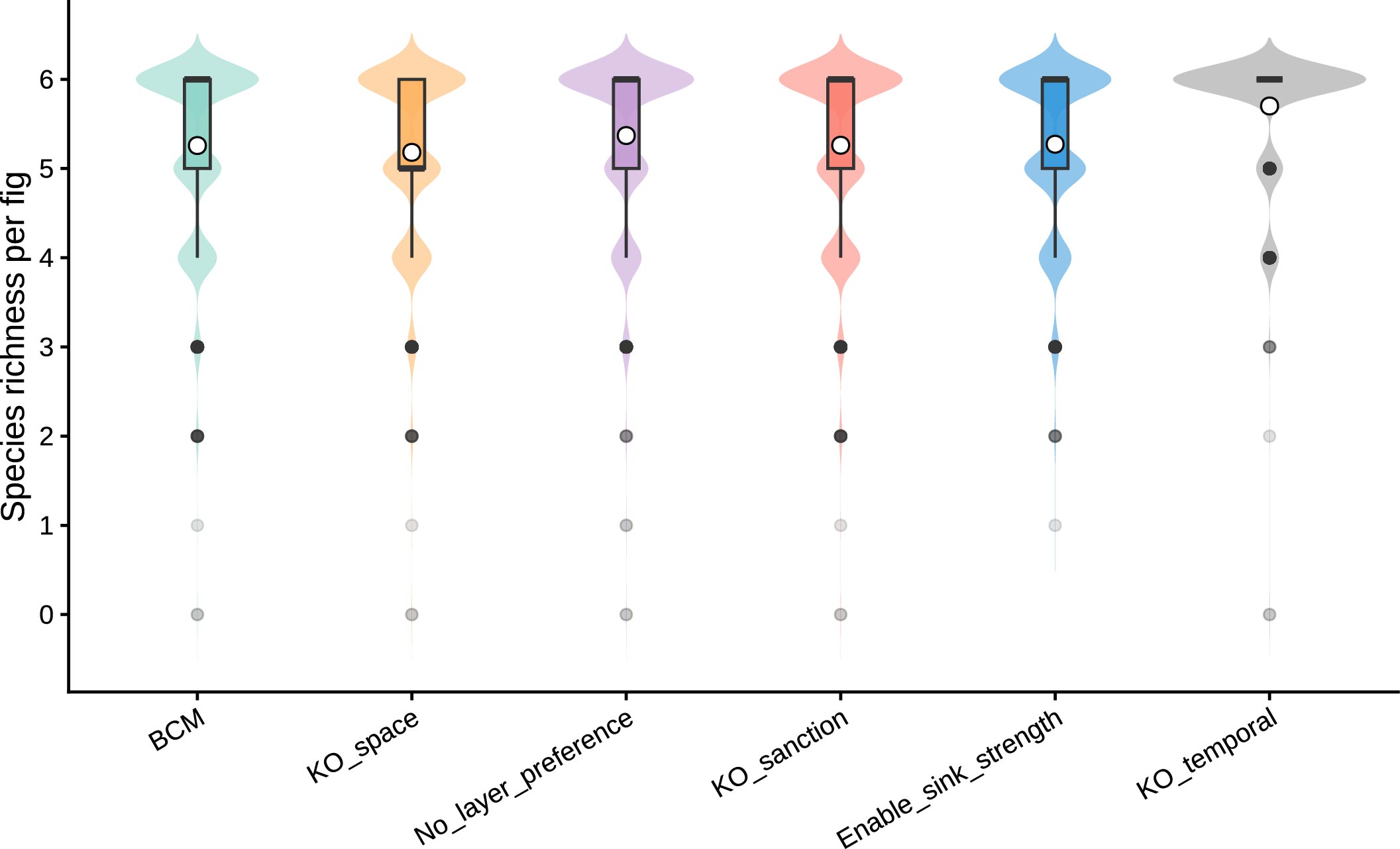

Supplement: Supplementary file 5 — Figure S5: Distributions of species richness per fig across mechanism‐altered simulations. [file ECE3-16-e74018-s006.jpg]
