# Supplementary material for: figsimR: An R Package for Simulating Fig–Wasp Community Dynamics
Source: Ecol Evol. 2026 Jul 20;16(7):e74018. doi: 10.1002/ece3.74018 (PMC13385217; doi:10.1002/ece3.74018)
Supplement: Supplementary file 6 — Table S1: Full parameter list and their descriptions in the figsimR package. [file ECE3-16-e74018-s005.docx]

**Table S1.** Full parameter list and their descriptions in the figsimR package.

| **Parameter** | **Scope** | **Default** | **Description** |
| --- | --- | --- | --- |
| alpha | global | 1.3 | Numeric. Exponent in the power-law relationship between fig diameter and flower number. Empirically estimated around 1.2-1.4 for many figs. |
| drop_cancels_emergence | global | FALSE | Logical. If FALSE, dropped figs produce zero emergence and seeds. If TRUE, their output remains. |
| egg_success_prob | global | NULL | Named numeric vector. Global probability that a single oviposition attempt results in a successful egg. Applies when use_egg_success_by_phase = FALSE. |
| egg_success_prob_by_phase | phase | NULL | Nested list. Overrides global success probabilities by entry phase. A species-level list of phase-specific reproductive-success probabilities. For each species and developmental phase, the supplied value is used as the probability that an arriving or oviposition-attempting female contributes eggs during that phase. If no phase-specific value is supplied, the model uses the baseline egg_success_prob. Realized egg numbers are subsequently determined by fecundity, host availability, ovary-layer accessibility, and resource constraints. Format: species_name = list(phase1 = 0.5, phase2 = 0.7,...). |
| enable_drop | global | TRUE | Logical. Enables fig abortion due to host sanctions. If flower use proportion exceeds host_sanction, fig may abort. In the output “drop_prob” column: Ranges from 0 (never drop) to 1 (always drop); “is_dropped” column: no drop (0) and drop (1). |
| entry_distribution | species | “lognormal” | Character. Either “nb” (negative binomial) or “lognormal”.  Defines how per-species entry numbers are simulated:  “nb”: entries are drawn from NB( mu = entry_mu, size = entry_size ). “lognormal”: log(entry_mu) - 0.5 used as meanlog; sdlog = 1 / sqrt(entry_size); result used as Poisson rate. |
| entry_mu | species |  | Named numeric vector. Mean number of individuals of each species attempting to “enter” (arrival or oviposition-attempt events) each fig. Acts as expected value for entry distribution. |
| entry_priority | phase |  | Named list. Defines the temporal entry order of species. Each element is a phase (e.g., phase1), with a vector of species names. Earlier phases enter first and may influence resource or host availability for later phases. |
| entry_size | species |  | Named numeric vector. Controls variation in entry distribution (the dispersion of the arrival or oviposition-attempt distribution):  For entry_distribution = “nb”: used as NB size parameter. For “lognormal”: used to derive sdlog = 1 / sqrt(entry_size). |
| fecundity_dispersion | species |  | Named numeric vector. Dispersion parameter for fecundity (size in NB distribution). Controls variability in egg output across individuals. |
| fecundity_mean | species |  | Named numeric vector. Mean number of eggs laid per female individual, per species. Used as mu in a negative binomial distribution. |
| fig_diameter_max | global | 4 | Maximum fig diameter used to cap values (optional). |
| fig_diameter_mean | global | 2.5 | Numeric. Mean fig diameter (in cm). Used to generate per-fig flower number via the formula: flower_count = k, diameter^alpha, Gamma(shape = 20, scale = 0.1). Larger diameters yield more ovules. |
| fig_diameter_min | global | 1.3 | Numeric. Truncate simulated diameters (and thus flower counts) to a biologically reasonable range. |
| fig_diameter_sd | global | 1.2 | Numeric. Standard deviation of fig diameter. Each fig’s diameter is drawn from a normal distribution with this mean and SD. |
| host_sanction | global | 0.8 | Numeric [0,1]. Threshold parameter used in the optional host-sanction/fig-abortion module. Threshold of flower use (e.g., 0.8). If exceeded, the fig may abort (drop) due to excessive exploitation by wasps. When enable_drop = TRUE, fig drop probability is calculated as a logistic function of resource_ratio relative to this threshold. This parameter does not directly represent the probability of abortion, and it is retained as an optional alternative fig-retention rule. |
| interaction_matrix | species | NULL | Optional numeric matrix. Square matrix defining pairwise interspecific interactions. Each entry affects the entry probability of other species via: adjusted_mu = entry_mu exp(interaction_weight interaction_sum). |
| interaction_weight | species | 0 | Numeric. Controls strength of interspecific interactions as defined in interaction_matrix. 0 means no effect. |
| k | global | 300 | Numeric. Scaling constant for estimating flower number from fig diameter. Represents average number of flowers per cm^ alpha. |
| layer_preference | species | NULL | Named list. For each species, a named numeric vector giving probabilities of oviposition in core, mid, and outer ovary layers. Sum must be 1. Or “none” is not given species-specific layer preferences; their oviposition attempts are allocated according to remaining ovary-layer availability. Used only if use_layering = TRUE. |
| lhs_param_list | global |  | List of input parameters (entry, fecundity, etc.). |
| max_entry_table | species | NULL | Named numeric vector. Maximum number of individuals of each species that can “enter” (arrival or oviposition-attempt) a fig, scaled by fig size. Used to truncate simulated entry counts. |
| num_figs | global | 1000 | Integer. Number of figs to simulate in each replicate. Default = 1000. |
| p_no_entry | global | 0.002 | Numeric [0,1]. Probability that a fig receives no wasp entries. Simulates failed colonization events. |
| p_pollination_per_ovule | global | 0.98 | Numeric [0,1]. Probability that each unused ovule becomes a viable seed, representing background pollination efficiency. |
| param_list | global |  | A named list of species parameters used in the fig wasp simulator. Must include at least: entry_mu, entry_size, fecundity_mean, fecundity_dispersion, and egg_success_prob. Optional entries include layer_preference, parasitism_prob, and egg_success_prob_by_phase. |
| parasitism_prob | species | NULL | Named numeric vector. In the default layered interaction model, parasitoid reproduction is constrained by defined host availability and ovary-layer availability rather than by this probability parameter. Used only if use_supplemental_parasitism = TRUE. |
| record_individual | global | FALSE | Logical. If TRUE, the function returns an additional element individual_eggs, which records per-individual oviposition events (vector of eggs per female). |
| species_list | global |  | Character vector of species names (used to extract relevant columns). |
| species_roles | species |  | List. Defines the ecological roles and interactions of all species. Contains three components: guild: character vector assigning each species to “pollinator”, “galler”, or “parasitoid”. hosts: list mapping parasitoids to their hosts. parasitoid: list mapping hosts to their attacking parasitoids. This structure defines trophic constraints (e.g., parasitoids cannot oviposit unless their hosts are present). |
| use_egg_success_by_phase | phase | TRUE | Logical. Use egg success probabilities by phase. Default TRUE. |
| use_flower_limit | global | TRUE | Logical. If TRUE, clamps fig diameter between fig_diameter_min and fig_diameter_max before calculating flower number. |
| use_layer_preference | species | TRUE | Logical. If TRUE, species will preferentially oviposit in specific layers as defined in layer_preference. |
| use_layering | species | TRUE | Logical. If TRUE, fig flowers are divided into spatial layers (core, mid, outer). |
| use_supplemental_parasitism | species | FALSE | Logical. Whether to activate supplemental parasitism. Default FALSE. |
| use_sink_strength | global | FALSE | Logical. If TRUE, compute sink-strength metrics but  do NOT alter legacy drop decision. Adds columns: sink_strength, sink_prop, sink_below_min, sink_above_max. Segar, Simon T., Sotiria Boutsi, Daniel Souto-Vilaros, Martin Volf, Derek W. Dunn, Astrid Cruaud, Rodrigo AS Pereira, Jean-Yves Rasplus, and Finn Kjellberg. “The diversity of *Ficus*.” Annals of Botany (2025): mcaf280. |
| sink_w_gall | global | 1 | Numeric [0,1]. Per-ovule sink weight for galled ovules (default 1.0). |
| sink_w_seed | global | 1.5 | Numeric [0,1]. Per-ovule sink weight for seeds (default 1.5). |
| sink_linear_coef | global | 1 | Numeric [0,1]. Global multiplier on sink strength (default 1.0). |
| sink_min_prop | global | 0.2 | Numeric [0,1]. Reference lower bound of sink proportion (default 0.20). |
| sink_max_prop | global | 0.95 | Numeric [0,1]. Reference upper bound of sink proportion (default 0.95). |
