# Supplementary material for: figsimR: An R Package for Simulating Fig–Wasp Community Dynamics
Source: Ecol Evol. 2026 Jul 20;16(7):e74018. doi: 10.1002/ece3.74018 (PMC13385217; doi:10.1002/ece3.74018)
Supplement: Supplementary file 7 — Table S2: Initial estimates and final optimized parameters in the Baseline Configuration Modeling (BCM). Initial values were informed by literature and biological reasoning. Final values reflected results from the Latin Hypercube Sampling optimization procedure and were used in all subsequent simulations in the BCM. [file ECE3-16-e74018-s004.docx]

**Table S2.** Initial estimates and final optimized parameters in the Baseline Configuration Modeling (BCM). Initial values were informed by literature and biological reasoning. Final values reflected results from the Latin Hypercube Sampling optimization procedure and were used in all subsequent simulations in the BCM.

| **Parameter** | **Species** | **Initial value** | **Optimized value** |
| --- | --- | --- | --- |
| entry_mu |  |  |  |
|  | Sycophaga testacea | 6 | 6.87 |
|  | Apocrypta sp. | 10 | 12.23 |
|  | Sycophaga mayri | 9 | 7.97 |
|  | Ceratosolen sp. | 13 | 11.25 |
|  | Sycophaga agraensis | 6 | 5.35 |
|  | Apocrypta westwoodi | 6 | 6.66 |
| entry_size |  |  |  |
|  | Sycophaga testacea | 9 | 8.6 |
|  | Apocrypta sp. | 8 | 9.42 |
|  | Sycophaga mayri | 9 | 10.43 |
|  | Ceratosolen sp. | 8 | 8.9 |
|  | Sycophaga agraensis | 7 | 7.9 |
|  | Apocrypta westwoodi | 7 | 8.4 |
| fecundity_mean |  |  |  |
|  | Sycophaga testacea | 22 | 21.3 |
|  | Apocrypta sp. | 4 | 3.4 |
|  | Sycophaga mayri | 21 | 19.5 |
|  | Ceratosolen sp. | 80 | 90.1 |
|  | Sycophaga agraensis | 3 | 3.4 |
|  | Apocrypta westwoodi | 5 | 4.3 |
| fecundity_dispersion |  |  |  |
|  | Sycophaga testacea | 1.5 | 1.7 |
|  | Apocrypta sp. | 2.2 | 2 |
|  | Sycophaga mayri | 1.5 | 1.5 |
|  | Ceratosolen sp. | 1 | 1.3 |
|  | Sycophaga agraensis | 1.5 | 1.7 |
|  | Apocrypta westwoodi | 1 | 1.3 |
| egg_success_prob |  |  |  |
|  | Sycophaga testacea | 0.8 | 0.86 |
|  | Apocrypta sp. | 0.6 | 0.44 |
|  | Sycophaga mayri | 0.6 | 0.52 |
|  | Ceratosolen sp. | 0.9 | 0.65 |
|  | Sycophaga agraensis | 0.4 | 0.38 |
|  | Apocrypta westwoodi | 0.6 | 0.42 |
| egg_success_prob_by_phase | |  |  |
|  | Sycophaga testacea | phase1 = 0.8 | phase1 = 0.68 |
|  | Apocrypta sp. | phase1 = 0.4, phase2 = 0.4 | phase1 = 0.43, phase2 = 0.34 |
|  | Sycophaga mayri | phase2 = 0.9 | phase2 = 0.72 |
|  | Ceratosolen sp. | phase2 = 0.8 | phase2 = 0.66 |
|  | Sycophaga agraensis | phase2 = 0.1, phase3 = 0.8 | phase2 = 0.13, phase3 = 0.7 |
|  | Apocrypta westwoodi | phase1 = 0.4, phase2 = 0.4 | phase2 = 0.09, phase3 = 0.61 |
| layer_preference |  |  |  |
|  | Sycophaga testacea | core = 0.7, mid = 0.25, outer = 0.05 | core = 0.89, mid = 0.21, outer = 0.05 |
|  | Apocrypta sp. | core = 0.05, mid = 0.1, outer = 0.85 | core = 0.05, mid = 0.08, outer = 0.93 |
|  | Sycophaga mayri | core = 0.4, mid = 0.4, outer = 0.02 | core = 0.38, mid = 0.46, outer = 0.02 |
|  | Ceratosolen sp. | core = 0.7, mid = 0.1, outer = 0.01 | core = 0.65, mid = 0.12, outer = 0.01 |
|  | Sycophaga agraensis | core = 0.01, mid = 0.5, outer = 0.49 | core = 0.01, mid = 0.39, outer = 0.56 |
|  | Apocrypta westwoodi | core = 0.01, mid = 0.5, outer = 0.49 | core = 0.1, mid = 0.6, outer = 0.6 |
| max_entry |  |  |  |
|  | Sycophaga testacea | 20 | 20 |
|  | Apocrypta sp. | 20 | 20 |
|  | Sycophaga mayri | 20 | 20 |
|  | Ceratosolen sp. | 20 | 20 |
|  | Sycophaga agraensis | 20 | 20 |
|  | Apocrypta westwoodi | 20 | 20 |
| parasitism_prob |  |  |  |
|  | Apocrypta sp. | 0.85 | 0.95 |
|  | Sycophaga agraensis | 0.9 | 0.82 |
|  | Apocrypta westwoodi | 0.6 | 0.46 |
